# Electrochemiluminescence Discloses Active Intermediates in Oxygen Evolution Reactions of Metal–Organic Framework

**DOI:** 10.1002/advs.202506309

**Published:** 2025-06-20

**Authors:** Xuan Chen, Bing‐Xin Sun, Wei Xu, Chen‐Hui Yin, Shi‐Yi Zhou, Yi‐Han Qian, Lu‐Nan Zhang, Qin Xu, Huan Pang, Cheng Ma

**Affiliations:** ^1^ School of Chemistry and Chemical Engineering Yangzhou University Yangzhou 225002 China

**Keywords:** electrochemiluminescence, in situ visualization, metal–organic framework, oxygen evolution reactions

## Abstract

In situ disclosure about the complex four‐electron transfer of anodic oxygen evolution reaction (OER) is crucial to further optimization of electrocatalysts. However, tracing the formation and evolution of unstable intermediates remains challenging. Here, electrochemiluminescence (ECL) analysis and imaging technique are developed to in situ monitor the important intermediate hydrogen peroxide intermediate (OOH*) on transition‐metal‐based 7,7,8,8‐tetracyanoquinodimethane (TM─TCNQ) metal–organic framework materials as OER catalysts. Thanks to the highly selective ECL probe L‐012 to OOH*, the ECL signal evolution (e.g., onset ECL potential and maximal ECL intensity) is bridged with the generation and accumulation of OOH*, allowing for convenient and quick judgment to the rate‐determining step of OER. Due to the spatial and temporal resolution of ECL microscopy, it is found that the phase I of Cu─TCNQ shows better catalytic activity. The COMSOL simulation also shows the heterogeneous current distribution during the OER reaction, in agreement with ECL imaging results. Finally, the ECL approach enables to compare the OER performance of three TM─TCNQ, well explaining the free energy profiles for different reaction intermediates. Therefore, the proposed ECL analysis and microscopy provide an alternative way to understand the detailed elemental steps in OER, thus further indicating the direction for high‐performance catalysts.

## Graphical abstract

Electrochemiluminescence microscopy is designed to monitor the important hydrogen peroxide intermediate on metal–organic framework during oxygen evolution reaction (OER) catalyzing. It creates a powerful and convenient analytical method to understand the detailed elemental steps, reveal the electrocatalytic activity of nanoparticle or spatial distribution of macroelectrode during OER, distinguish rate‐determining step, and guide to screen better OER catalysts.

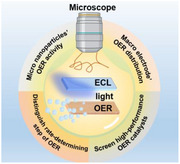

## 1 Introduction

Prior to the use of electrochemical water splitting or metal–air batteries as sustainable energy sources to partially replace fossil energy,^[^ ^1^ ^]^ there were anodic oxygen evolution reactions (OERs) that required high overpotential to overcome on account of slow kinetic.^[^ ^2^ ^]^ Therefore, in the past few decades, a large number of studies have been devoted to designing and synthesizing new anode materials to catalyze OER, hoping that some low‐cost and abundant materials have comparable or even more efficient catalytic activity than classic but low‐abundance precious metals.^[^ ^3^ ^]^ Among these new anode materials, transition‐metal (TM)‐based metal–organic framework (MOF) materials show great promise due to their large specific surface area, high porosity, and optimized active site distribution to maximize electron transfer and promote the mass transfer of reactants.^[^ ^4^ ^]^ Considering the high flexibility of forming the metal–ligand interface, the interfacial electron coupling interaction in MOF has been considered as one of the important factors to improve the redox reaction efficiency in the water splitting process,^[^ ^5^ ^]^ and the interfacial interaction is an important view to explore the catalytic redox reaction mechanism of MOF materials. Accordingly, the adsorbate evolution mechanism (AEM)^[^ ^6^ ^]^ with metal bands acting as the redox center is used to explain the complex four‐electron OER. However, the in situ adsorbate intermediates are difficult to be detected because of the short lifetime after separation from reaction sites.^[^ ^7^ ^]^ This means that we either need to use in situ equipment like electrochemical impedance spectroscopy,^[^ ^8^ ^]^ in situ FTIR spectra,^[^ ^9^ ^]^ rotating disk electrodes,^[^ ^10^ ^]^ liquid cell transmission electron microscopy,^[^ ^11^ ^]^ or electrical analysis methods and descriptors (polarization current curve and Tafel slope, etc.^[^ ^12^ ^]^) to study the OER mechanism.

Electrochemiluminescence (ECL) is a process in which electrochemical products undergo high‐energy electron transfer reactions to form excited luminescent emitters.^[^ ^13^ ^]^ It represents a clever combination of optical and electrochemical methods with unique advantage,^[^ ^14^ ^]^ whose advantages include high sensitivity and confinement effect from the electrochemistry and has a near‐zero background due to the absence of excitation light contrast to other in situ surface heterogeneity analysis techniques such as in situ Raman, surface enhanced infrared absorption spectroscopy. Moreover, ECL imaging technology can track the dynamic evolution of active sites or species in real time (such as valence state changes, intermediate adsorption/desorption), with a time resolution of up to the millisecond level. In virtue of the low lifespan of free radical coreactant, ECL also has a high spatial resolution to characterize the in situ analysis.^[^ ^15^ ^]^ Therefore, ECL, as a powerful analytical method, is now commercially available in therapeutic applications and clinical testing.^[^ ^16^ ^]^ Recently, the ECL technique has been widely used in immunoassays,^[^ ^17^ ^]^ bioimaging,^[^ ^18^ ^]^ and material analysis.^[^ ^19^ ^]^ Luminol, as a most common ECL luminophore, can emit ECL with reactive oxygen species (ROS) as coreactant. Therefore, some OER electrocatalysts can enhance the ECL of luminol at the anode by accumulating ROS through OER.^[^ ^20^ ^]^ By contrast, the anodic ECL of Ru(bpy)_3_^2+^ can be inhibited by OER due to the competitive relationship between OER and ECL reactions.^[^ ^21^ ^]^ In terms of mechanism studies, the ECL technique has good compatibility with interfacial electrocatalytic reactions.^[^ ^22^ ^]^ As a result, the ECL signal can in turn reflect the OER performance of electrocatalysts, such as the stepwise heterogeneous electrocatalytic responses^[^ ^23^ ^]^ and elementary reaction steps.^[^ ^24^ ^]^ However, the previous works took the four electrons transfer in OER as a whole and used the overall ECL strength as the output signal. These methods assumed that the step of generating hydrogen peroxide intermediate (OOH*) was the rate‐determining step (RDS) in the OER process.^[^ ^23^, ^24^, ^25^ ^]^ Taking the hypothesis that the formation of OOH* is the RDS during OER will misjudge the OER activity and RDS among different catalysts. Therefore, it is necessary to elucidate the correct rate‐determining step in the OER process by ECL method.

**Scheme 1:**
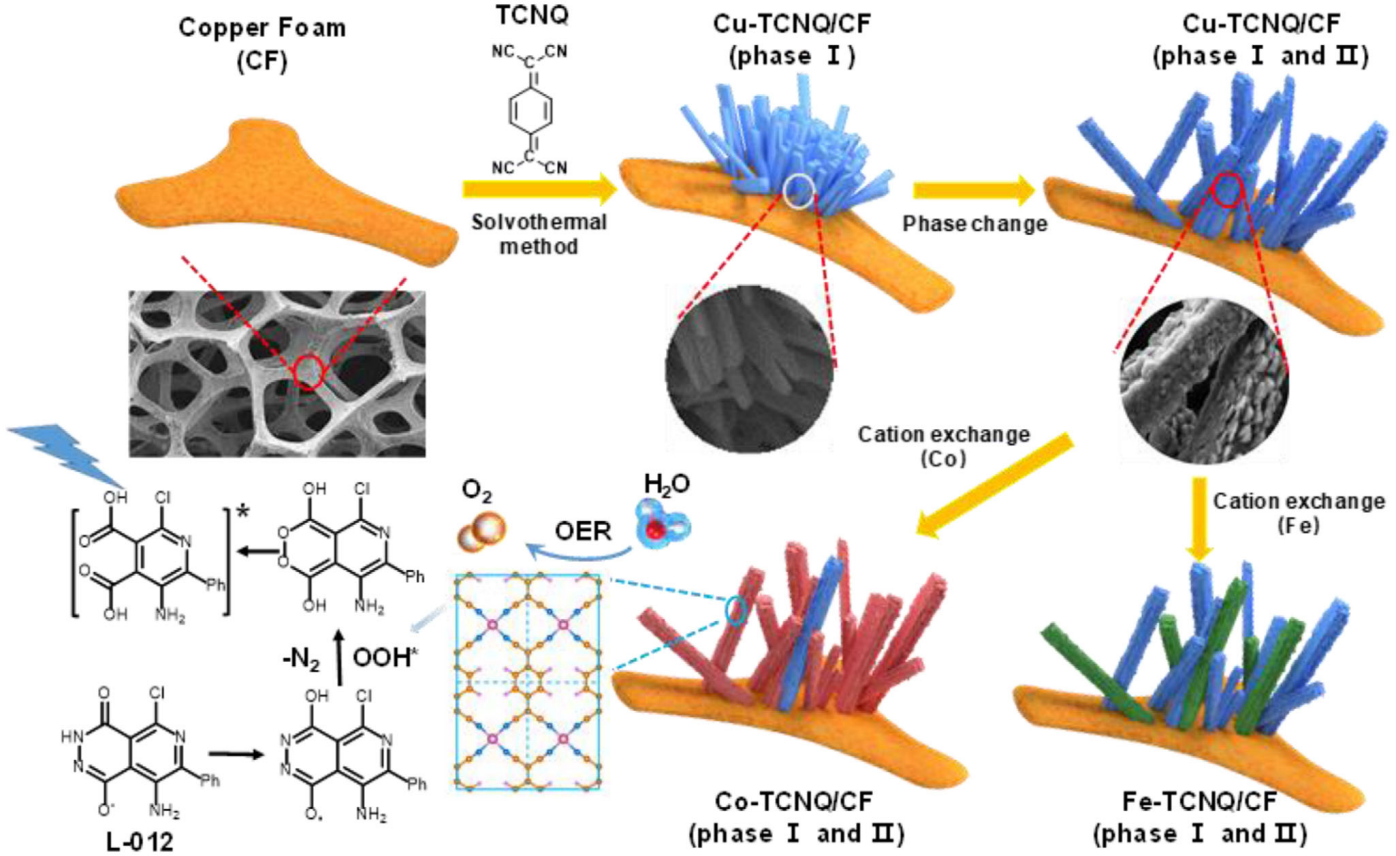
Schematic illustration of TM─TCNQ/copper foam (CF) preparation and the proposed mechanism for electrocatalytic hydroperoxide intermediate generation and ECL enhancement during OER.

In this work, we use 7,7,8,8‐tetracyanoquinodimethane (TCNQ) as the ligand and TM ions to synthesize TM─TCNQ MOF. TCNQ is known as one of the strongest organic electron acceptors (electron affinity 2.88 eV) and has long been widely considered as a key candidate for the formation of semiconductor organic/inorganic charge transfer compounds.^[^ ^26^ ^]^ TM─TCNQ MOF not only inherits the high conductivity of TCNQ in the aqueous phase but also has the 2D TM─N_4_ sheet skeleton that can be firmly anchored to various monometal atomic sites in the pores. Then, we use luminol derivate L‐012 as the ECL luminophore to study the OER performance of TM─TCNQ MOF (**Scheme** **1**). L‐012 emits anodic ECL that can be enhanced by peroxide adsorbate intermediates produced during the OER process. When L‐012 oxidation products react with adsorbed OOH* in the catalytic site of MOF, the excited state L‐012 is generated. Therefore, the ECL signal reveals the important intermediate OOH* in the OER process. Different from other in situ analysis methods, we converted the short‐life OOH* into easily measured ECL signals that provide more detailed information in the four‐electron OER. Our results demonstrated that direct visualization of electrocatalytic reactions at macroelectrode or microparticles were achieved via ECL microscopy to characterize electrocatalytic performance. In addition, compared with prior works, detailed electrocatalytic information on the elementary reaction step, especially the rate‐determining step, was obtained by two parameters from ECL, including the sequence of kinetics (*η*_TM‐ECL_) and the difficulty of thermodynamic accumulation (*I*_TM‐ECL_). It offers a new and convenient approach to screen and optimize OER catalysts.

## 2 Results and Discussion

**Figure 1:**
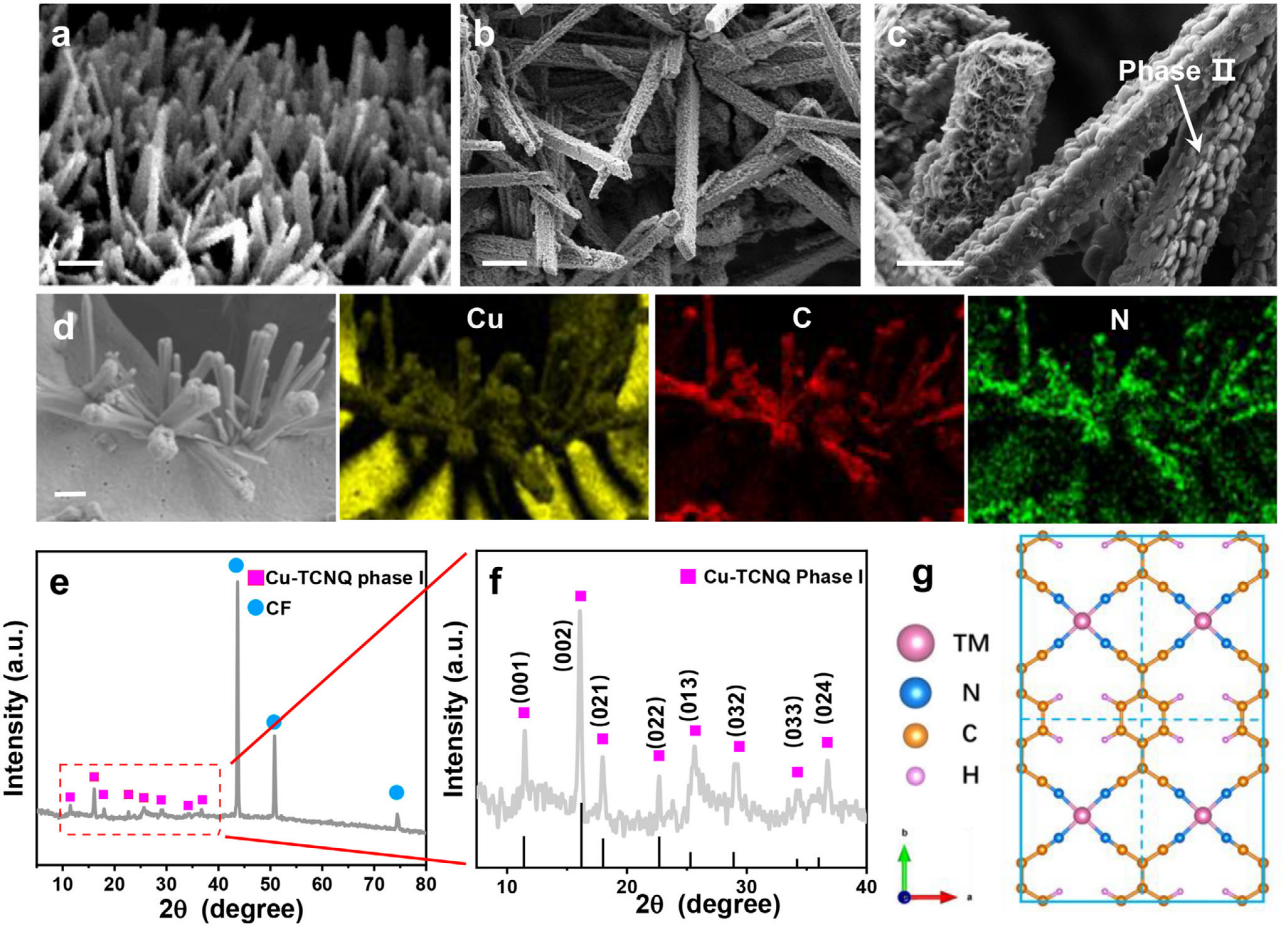
SEM images of Cu─TCNQ/CF a) after solvothermal method for 5 min, b) lengthened immersion time to phase change for 3 h, and c) its enlarged image. Scale bar: 2 µm. d) Elemental mapping images of Cu, C, N for Cu─TCNQ/CF. The shadow is attributed to the needle‐like structures blocking the electron beam. Scale bar: 2 µm. e) XRD pattern of Cu─TCNQ/CF and f) its enlarged image. g) The optimized geometry of TM─TCNQ nanosheet (TM = Fe, Co, Cu, etc.) monolayer in 2 × 2 supercell.

Cu─TCNQ MOF was in situ grown on copper foam (CF) substrate by hydrothermal solution method. The copper foam provided a copper source that reacted with saturated TCNQ solution to form Cu─TCNQ MOF. As shown in **Figure** **1**a, the scanning electron microscope (SEM) images of Cu─TCNQ clearly showed the closely packed needles morphology with sharp edges, the average length and width of these needles were measured as 8–10 and 0.4–0.6 µm, respectively.^[^ ^27^ ^]^ Such needle structures of Cu─TCNQ are suggested as kinetically favored structures of phase I.^[^ ^28^ ^]^ Although phase I of Cu─TCNQ possessed good conductivity, the specific surface area and stability of phase I for OER was not as good as phase II of Cu─TCNQ. Therefore, to partially transform phase I to phase II, the needle structures of Cu─TCNQ phase I were immersed in acetonitrile solution for different times. With time elapsing, the needle structures of Cu─TCNQ phase I gradually transformed into square tubes covered by platelet shape (phase II) due to the reformation of dissolving Cu─TCNQ (Figure S1, Supporting Information).^[^ ^29^ ^]^ Finally, the immersion time of 3 h was chosen since the structure of platelet‐morphology (phase II)‐covered square tube (phase I) (Figure 1b,c) was reported with the highest catalytic activity for OER.^[^ ^30^ ^]^

The energy‐dispersive X‐ray elemental mapping images (Figure 1d) confirmed the presence of Cu, C, and N elements in Cu─TCNQ/CF and these elements dispersed uniformly. The XRD pattern showed the main component of the two‐phase composite was still phase I (Figure 1e,f). Although Cu─TCNQ phase II was the thermodynamically favored structure at the recombination stage, the XRD peaks of platelet morphology (phase II) were too weak to be detected at this stage because of the low proportion of phase II in the Cu─TCNQ catalyst.

Accordingly, we designed the composite structure that included tube‐shaped Cu─TCNQ (phase I) as a conductive substrate and superficial platelet‐shaped Cu─TCNQ (phase II) nanosheets as active sites for OER. To further characterize the component of Cu─TCNQ, we used X‐ray photoelectron spectroscopy (XPS). As shown in Figure S2a (Supporting Information), the spectrum of Cu─TCNQ indicated Cu, C, and N elements in the material, demonstrating all necessary elements existed in Cu─TCNQ. Figure S2b (Supporting Information) showed the fine XPS spectrum of Cu 2p of Cu─TCNQ.^[^ ^31^ ^]^ In the C 1s region (Figure S2c, Supporting Information), the binding energies at 284.7 and 286.1 eV corresponded to carbon bonds and the CN group in TCNQ, respectively. In addition, a shake‐up satellite (identified as Sat.) at 288.1 eV was due to the unsaturated carbon bonds. In Figure S2d (Supporting Information), N 1s binding energies occurred at ≈398.5 eV, along with a shake‐up feature at 399.8 eV. Taken together, all the above characterizations manifest the successful synthesis of the Cu─TCNQ catalyst.

Then, we investigated the OER property of Cu─TCNQ as an electrocatalyst. Compared with bare CF substrate, Cu─TCNQ showed less overpotential and higher oxidation current in linear sweep voltammetry (LSV) curves (Figure S3, Supporting Information), suggesting the good OER performance of Cu─TCNQ. However, traditional LSV curves give information about the overall OER activity of electrocatalysts rather than the elementary steps of the four‐electron transfer process. To address this problem, we wanted to use an ECL probe to investigate the more detailed process described in the Equations (1)–(4). Among the mechanisms of OER under alkaline conditions, the adsorption theoretical model has been widely accepted, and the detailed reaction mechanism was shown by the following equations, where, e.g., OH*, O*, OOH* denote hydroxyl radical, oxygen radical, and hydrogen peroxide radical adsorption intermediates, respectively^[^ ^32^ ^]^

(1) $$OH^{-}+\mathrm{Substrate}\overset{O}{\to }H^{*}+e^{-}$$

(2) $$OH^{-}+OH^{*}\overset{O^{*}}{\to }+H_{2}O+e^{-}$$

(3) $$O^{*}+OH^{-}\overset{\mathrm{OO}}{\to }H^{*}+e^{-}$$

(4) $$\mathrm{OO}H^{*}+OH^{-}\overset{O_{2}}{\to }\left(\mathrm{gas}\right)+H_{2}O+e^{-}$$

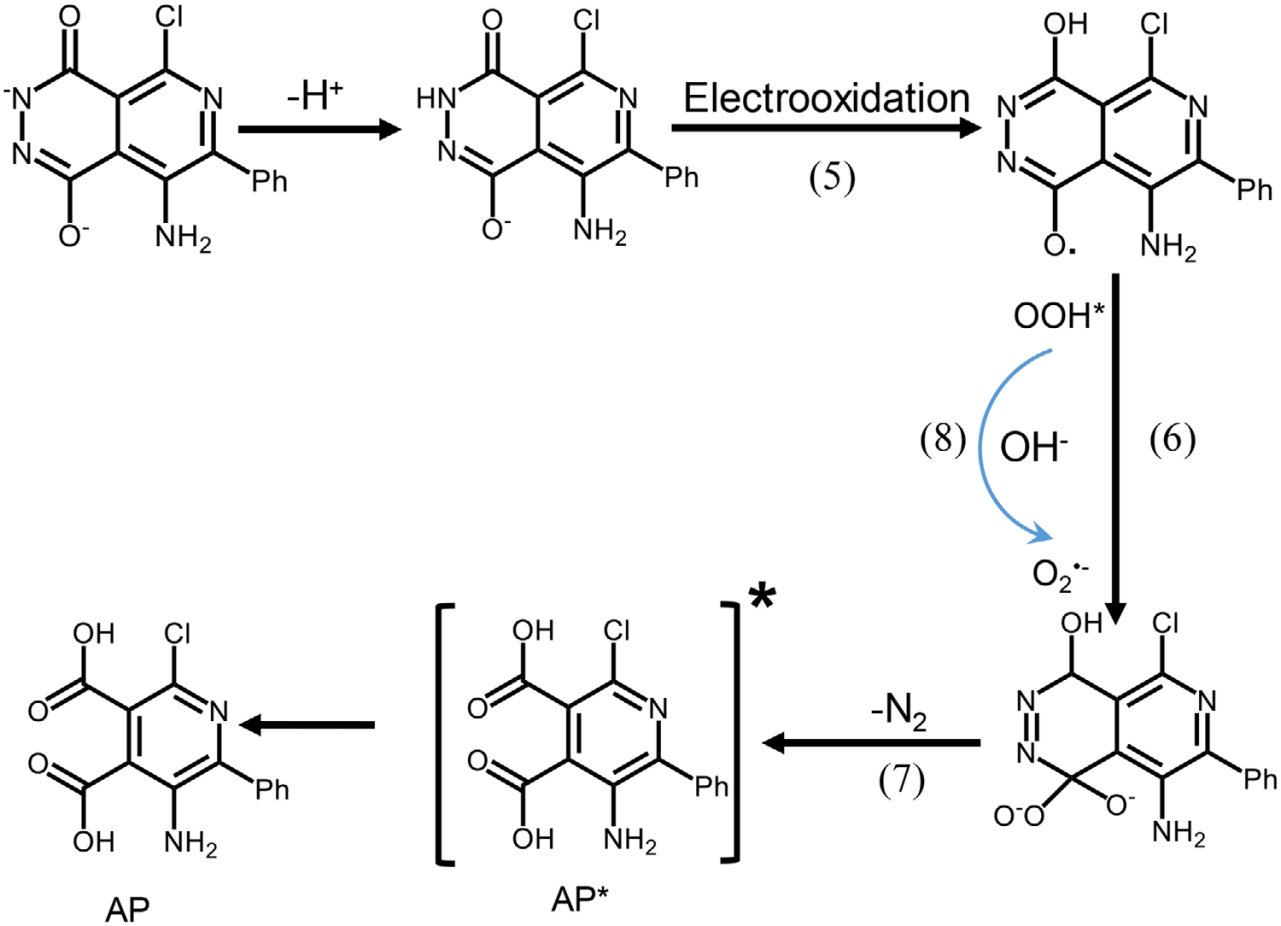

L‐012 is a luminol derivative with superior ECL efficiency in the presence of ROS as coreactants. Taking OOH* as an example of ROS, the ECL reactions involving L‐012 and OOH* were described in Equations (S5)–(S8). In the presence of OOH*, the ECL intensity of L‐012 could be enhanced significantly due to the cascade reactions between OOH* and L‐012.^[^ ^24^ ^]^ Coincidentally, OOH* is also an important intermediate during electrochemical OER on the catalyst surface. Therefore, the ECL intensity is positively related to the amount of OOH* generated on the surface of Cu─TCNQ. We expected that the ECL emission of L‐012 could quantitatively monitor OOH* during OER processes in real time.

**Figure 2:**
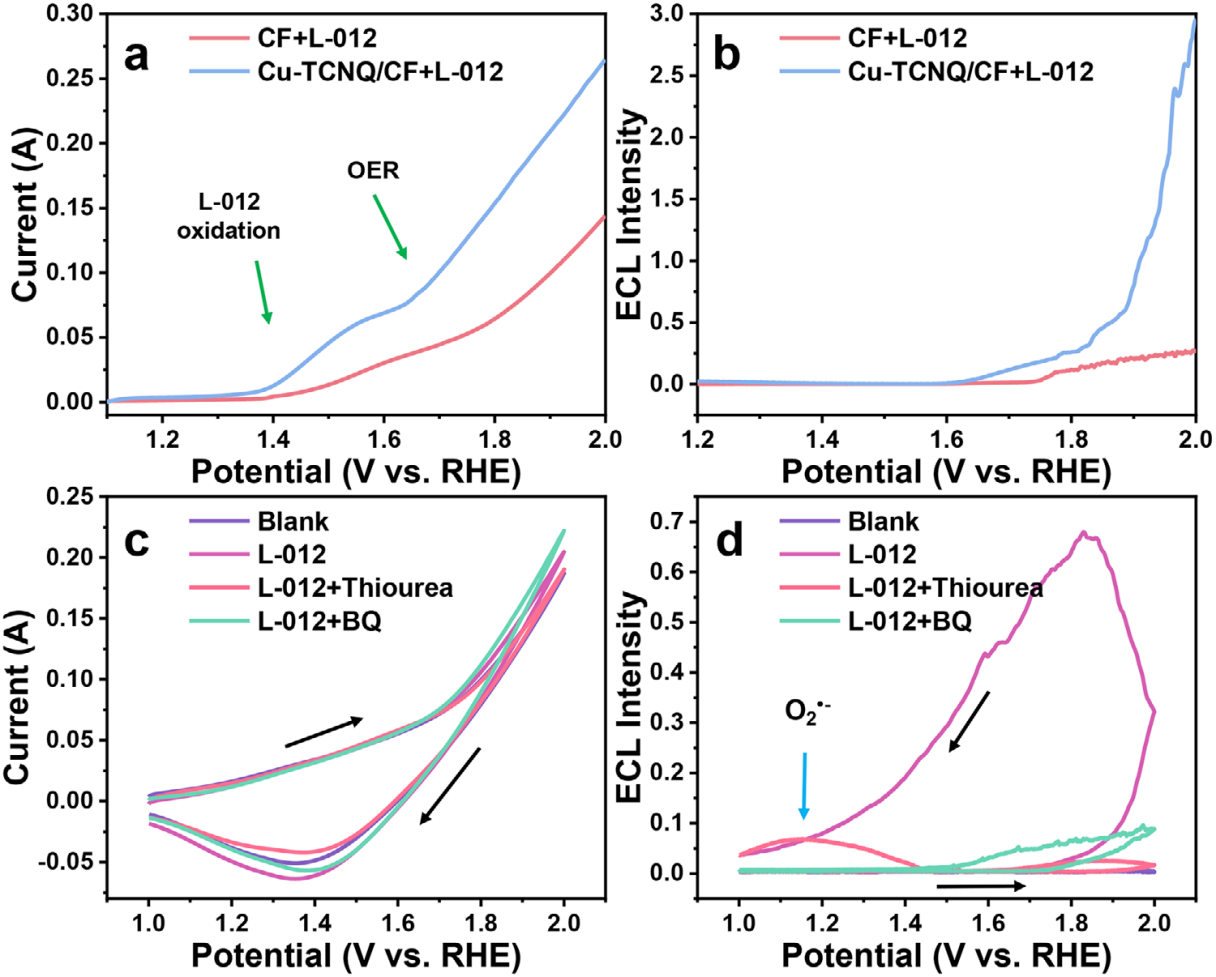
a) LSV curves and b) *I*_ECL_–potential curves of CF and Cu─TCNQ/CF with the addition of 1 mm L‐012 in 1.0 m KOH. c) CV curves and d) *I*_ECL_–potential curves of Cu─TCNQ/CF with the addition of 100 µm L‐012 and 0.1 mm BQ or 10 mm thiourea as radical scavengers in the blank electrolyte (1.0 m KOH). Scan rate: 0.1 V s^−1^.

To verify this hypothesis, we recorded the LSV curves and the corresponding ECL intensity curves with potential sweeping (**Figure** **2**a,b). The onset of the oxidation current of L‐012 appeared at 1.3 V (vs RHE), which was earlier than that of OER (1.63 V vs RHE). Because the applied voltage was not enough to drive OER and produce any ROS at this stage (below 1.63 V), the ECL signal remained very weak because ECL reactions rely on the generation of ROS (Equation (S6)). With the rise in applied potential, Cu─TCNQ on electrode surface began to catalyze OER along with the four‐electron transfer process. Thus, the oxidation current significantly rose beyond 1.63 V. Because OER generated OOH* on the surface of Cu─TCNQ, the ECL intensity also largely increased beyond 1.7 V, following the reaction mechanism described in Equations (S5),(S7). The peak of ECL spectrum was at 445 nm and consistent with characteristic spectrum of L‐012 (Figure S4, Supporting Information). By contrast, the blank copper foam substrate only showed very weak ECL emission due to its poor OER catalytic activity. The cyclic voltammetry (CV) and ECL–potential curves demonstrated that ECL emission was closely related to the ROS generated on the Cu─TCNQ surface during OER.

Because many oxidant intermediates generated during the OER process can react with L‐012 and enhance the ECL signal (Equations (1)–(4)), we tried to verify the ECL enhancement mechanism during Cu─TCNQ catalyzing OER. Considering that these intermediates (e.g., OH*, O*, OOH*) can be regarded as free radicals, we used free radical scavengers in the ECL system. As shown in Figure 2c,d, we recorded the cyclic voltammetry curves and ECL–potential curves of Cu─TCNQ in the solution containing either thiourea (OH• scavengers) or benzoquinone (BQ, O_2_^•−^ scavengers). Both thiourea and BQ largely decreased the ECL intensity, while the current in CV curves remained mostly stable after the addition of thiourea and BQ. The thiourea‐induced ECL decline indicated that OH*, as an important precursor to generate OOH*, was significantly reduced by the thiourea scavenger, thus indirectly decreasing the ECL intensity of L‐012. In addition, adsorption intermediate OOH* can spontaneously transfer into O_2_^•−^ radicals under alkaline conditions due to a Fenton‐like reaction (Equation (S8)).

However, these generated O_2_^•−^ radicals were immediately consumed in the presence of BQ scavenger. It broke the chemical equilibrium of Equation (S8) and decreased the content of OOH*. Therefore, the ECL intensity was significantly reduced in the presence of BQ as well. It is worth noting that BQ made the ECL peaks (around 1.2 V vs RHE) less than that in thiourea (indicated by the blue arrow). Because the ECL peak occurred at sweeping back, we believed the ECL peak was caused by the reduction reaction of O_2_ to O_2_^•−^ radicals. However, BQ specifically quenched O_2_^•−^ radicals and made ECL intensity significantly low. The above free radical scavenger experiments showed that the ECL signal of L‐012 had a high sensitivity to OOH* produced by OER.

In addition to the radical scavenger experiments, we further verified our theoretical mechanism and related conjecture by halting the applied potential when scanned to a specific potential. As shown in Figure S5a,b (Supporting Information), when the potential swept to 2 V, we halted the applied potential and recorded the following ECL trajectory. We found the ECL signal continuously intensified even though the potential was halted at 2 V. The long persistent luminescence of Cu─TCNQ was attributed to the porous nature of MOF materials. It made L‐012 take more time to diffuse into Cu─TCNQ and react with the OOH* or O_2_^•−^ in the pores.^[^ ^33^ ^]^ Moreover, the lifetime of O_2_^•−^ (about 60 s) is long enough to react with L‐012 to generate L• and further AP*, like the path of chemiluminescence, after the voltage is halted. When the potential was swept back to 1.6 V and then halted, the halted potential did not significantly influence the following afterglow ECL trajectory compared with the normal ECL–potential curve. The ECL intensity only slightly decreased because no superoxide free radicals were generated when halted at 1.6 V retrace. The phenomenon was consistent with the results of the above free radical scavenger experiments. In addition, the CV curves of Cu─TCNQ catalyzing OER remained very stable after 50 scan cycles, and the corresponding ECL–potential curves also remained unchanged (Figure S5c,d, Supporting Information). It proved not only the good stability of Cu─TCNQ as an OER catalyst but also the reliable ECL signal for evaluating OER activity.

**Figure 3:**
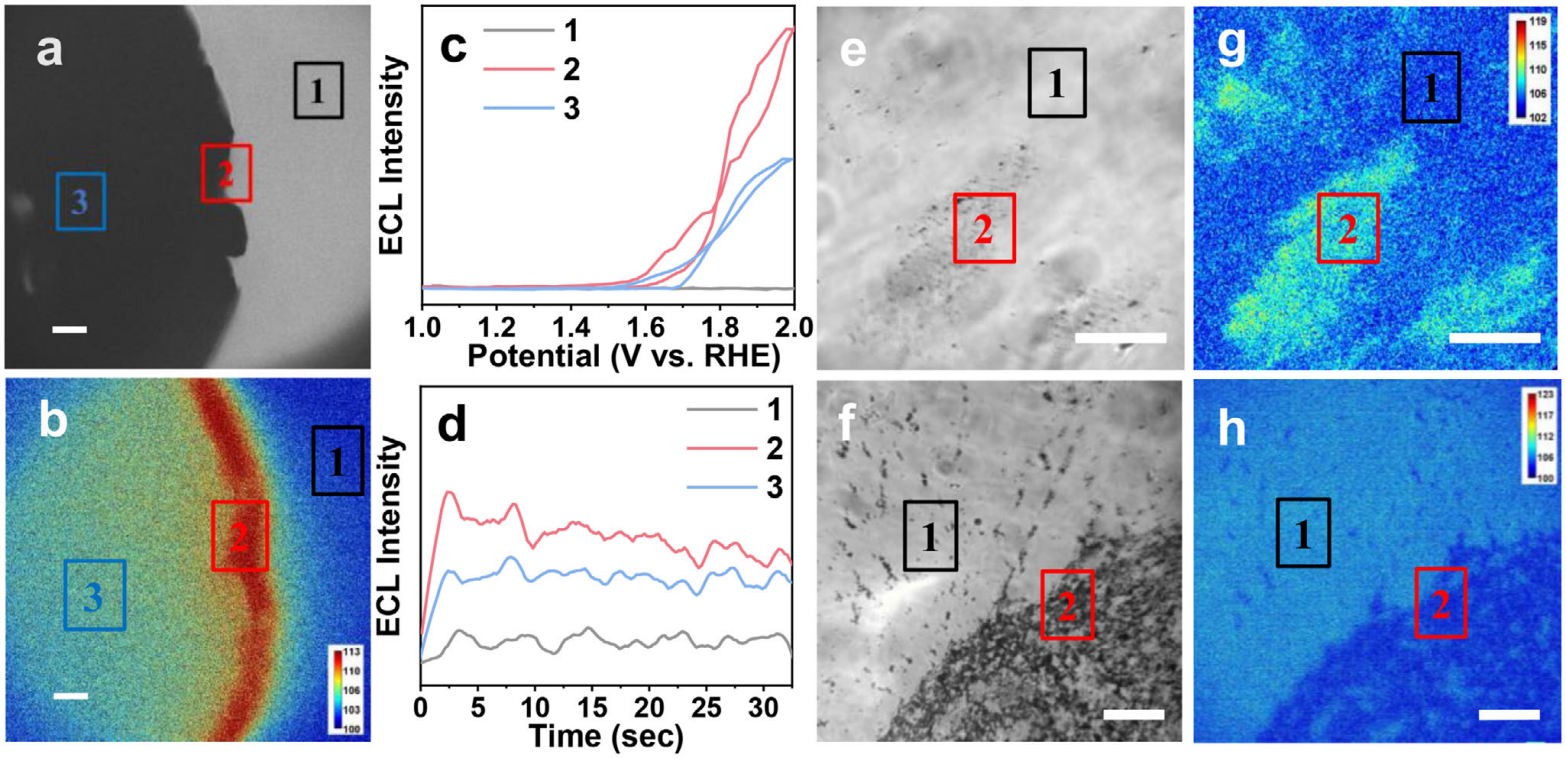
a) Bright field image and b) ECL image of Cu─TCNQ/CF electrode corner in 1 m KOH and 1 mm L‐012 electrolyte. The boxes 1, 2, and 3 represent the area of bulk solution, electrode edge, and electrode bulk. Exposure time: 200 ms. Scale bar: 50 µm. c) The profiles of ECL intensity of three box regions in (b) as a function of potential (cyclic voltammetry scan; scan rate, 0.1 V s^−1^). d) ECL intensity of three box regions in (b) as a function of time during a constant potential (2.0 V). Bright field images of ITO electrode loaded by Cu─TCNQ particles scraped from e) the edge and f) the bulk of Cu─TCNQ/CF electrode. g,h) The corresponding ECL images of (e, f) at the potential *E* = 2 V in 1 m KOH and 1 mm L‐012 electrolyte. Boxes of 1 and 2 represent the area without and with particle loading. Exposure time: 500 ms. Scale bar: 20 µm.

Considering that the prepared Cu─TCNQ consisted of two different phases (phase I and phase II), it is necessary to investigate the OER activity of the two phases, respectively. Thanks to the specific response of the ECL signal to OER, it enabled us to image the OER activity of two phases by ECL microscopy with high spatial resolution. Here, we focused on the end of the Cu─TCNQ electrode, whose bright field image was shown in **Figure** **3**a. When the voltage was applied to the Cu─TCNQ/CF electrode, we recorded the corresponding ECL image by microscope and differentiated OER activity based on the variation of ECL signal. Moreover, the rapid collection of ECL images during CV scan enables us to continuously monitor the ECL evolution under different voltages on electrode surface (Figure S8 and Movie S1, Supporting Information). In detail, the ECL image showed that the edge of Cu─TCNQ/CF emitted brighter ECL signal than the bulk of Cu─TCNQ/CF (Figure 3b). Figure 3c presented the profiles of ECL intensity for three box regions in Figure 3b under CV scan (1.0–2.0 V vs RHE). No distinct emission from the electrode was observed until OER began. When the potential was over 1.6 V, ECL emission increased rapidly, perfectly matched the information given in the curve by the photomultiplier tube measurement (Figure 2).

The corresponding enhanced ECL signals of each box gave clearly visible information on the distribution of OER activity regarding space that the area of edge has better OER activity. The collected ECL intensity versus reaction time curve at constant potential *E* = 2 V was demonstrated in Figure 3d. In the first 3 s, ECL intensity at electrode showed a rapidly growing stage indicating the drastic production of OOH* during electrocatalytic OER. As reaction progressed from 3 to 30 s, ECL–time curve entered a quasiequilibrium region where the electrocatalytic reaction tended to become equilibrium. The different trends in the generation of intermediate at these boxes confirmed the visualization of instantaneous local electrocatalytic behavior at catalysts surface. Both scan methods suggested that the edge of Cu─TCNQ seems to prefer to generate OOH* intermediate during OER. To verify this hypothesis, we used SEM to character the structure on the Cu─TCNQ/CF edge and bulk. As shown in Figure S6a,b (Supporting Information), the body was covered with a large number of platelet‐like nanolayers, while the edge showed many rod‐shaped structures. The different morphologies may indicate the different phase structures. To prove this point, we measured XRD spectra of the bulk and edge of Cu─TCNQ, respectively. The XRD spectra (Figure S6c, Supporting Information) showed that the bulk of Cu─TCNQ was mainly composed of phase II, while the edge of Cu─TCNQ was mainly composed of phase I. As discussed above, the rod‐shaped Cu─TCNQ in the edge was the composition of phase I and phase II, but the XRD peaks of platelet morphology (phase II) were too weak to be detected.^[^ ^31^ ^]^ We also attempted to perform ECL imaging of Cu─TCNQ nanoparticles on ITO electrodes. Figure 3e,f was the bright field images of particles scraped from the edge and bulk of the Cu─TCNQ/CF electrode, respectively. We compared two particles (marked as boxes 2) with blank ITO substrate (marked as boxes 1) at the constant potential *E* = 2 V. The particles from the edge showed stronger ECL intensity than the ITO substrate, while the particles from the bulk showed weaker intensity, verifying the conclusion that edge with phases I and II had better OER activity than the bulk with phase II (Figure 3g,h). In detail, the phase I has high electrical conductivity due to the tight π–π (Figure S6d, Supporting Information) stacking and effective charge transfer (1–100 S cm^−1^), while the conductivity of phase II is as low as the insulator range in Figure S6e (Supporting Information) (<10⁻⁶ S cm^−1^).^[^ ^28^ ^]^ Therefore, the poor conductivity of phase II is not conducive to the ECL or OER reaction driven by electricity. Because the ECL intensity directly reveals the OER activity on electrode surface, the dark contrast of phase II on ITO electrode suggests that even the OER activity of bare ITO is better than that of phase II. By contrast, the good conductivity of phase I of Cu─TCNQ showed better OER activity than bare ITO electrode. Therefore, phase I showed a brighter ECL signal than ITO electrode in Figure 3g. Because of the spatiotemporal resolution of ECL microscopy, the OER activity from different phases is clearly shown through the image contrast.

To visualize the ECL enhancement caused by OER, we recorded the ECL images sequence under a constant voltage at *E* = 2 V (Figure S7, Supporting Information). During catalyzing OER, oxygen molecules were generated on the electrode surface. Once generated O_2_ molecules increase beyond the O_2_ saturated solubility, these O_2_ molecules will immediately nucleate and form O_2_ nanobubbles at the Cu─TCNQ/CF interface. Meanwhile, the evolution of oxygen bubbles also could be indicated by ECL. Figure S7c (Supporting Information) presented the higher oxygen bubble density at the edge of the mesh, which is attributed to its better OER activity that could be proved by ECL (Figure S6b, Supporting Information). It is worth noting that the interface of the electrode and bubbles still had stronger ECL intensity although these nanobubbles occupy reactive sites of electrode. It could be because the presence of a large amount of OH^−^ at the interface of the bubble^[^ ^34^ ^]^ and then reacting with OOH* to produce O_2_^•−^ with longer life (Equation (S8), Supporting Information). In addition, oxygen bubbles may produce various ROS at the electrode interface that led to stronger ECL intensity. It is the first time that electrogenerated microbubbles enhancing the ECL intensity of luminol is reported. Except for prior works that the enhancement at the interface between the statical bubble and electrode,^[^ ^34^, ^35^ ^]^ we further explore the influence factors, including the kinds of bubbles, the dynamical changes of the luminescent layer along with the bubble size, and the influence of the counter electrode. In Figure S10 (Supporting Information), the CO_2_ bubble could inhibit complex ROS secondary reaction, in contrast with the O_2_ bubble, and the interface of the micro‐CO_2_ bubble cannot enhance the ECL without H_2_O_2_ addition in deoxygenated electrolyte, although a large amount of OH^•^ at the interface. When the H_2_O_2_ was added in Figure S10c (Supporting Information), the ECL enhancement appeared at the interface. Meanwhile, the thicker luminescent layer pointed to the counter electrode, indicating the generation of long‐life O₂^•^⁻ for local pH changes caused by excessive OH^−^ at pH 7. Then, Figure S9 (Supporting Information) showed the development of OER generating bubbles was recorded by ECL microscope, verifying the interface enhancement effect can extend to further distances along the bubble, which may come from the high‐efficiency mass transfer characteristics of microbubbles to transfer the AP* emitter.

The one‐electron oxidation of OH^−^ to hydroxyls (OH^•^) could be significantly facilitated around bubbles but without ECL emission (Figure S10b, Supporting Information), demonstrating that OH^•^ cannot enhance the ECL of L‐012 effectively. From the classical mechanism explanation of luminol ECL, luminol must transform into endoperoxide (1, 2‐dioxocyclobutanedione, LO_2_^2−^) to emit light efficiently, whether it is traditional chemiluminescence or ECL. This step is the core link of energy release and photon generation. However, a single hydroxyl radical (OH^•^) itself cannot directly form an endoperoxide (LO_2_^2−^).^[^ ^36^ ^]^ Moreover, OH^•^ is added as a powerful electrophilic oxidant to the aromatic ring and then inhibits the emission of luminol. The last but not the least critical requirement is that the introduction of ECL luminophores does not significantly influence the OER. In general, introducing excessive extraneous substances may affect the OER kinetics. Nevertheless, the reference under hydrodynamic conditions showed that such above impact of OH^•^ to luminol was weak (*<*1%) in the presence of luminol up to 0.1 mm,^[^ ^37^ ^]^ in contrast with OER/ORR, especially in the kinetics‐controlled region. Such a phenomenon could be explained by the fact that an alkaline environment would weaken the adsorption strength of luminol on catalyst surfaces, thanks to an electric repulsion effect.^[^ ^38^ ^]^ Taking the concern about O₂^•^⁻ taking part in the ECL reaction into consideration, OOH* as adsorbed state is more stable than OOH• in aqueous solution.^[^ ^39^ ^]^ Meanwhile, the reaction coefficient ratio of OOH• transition to O₂^•^⁻ is 1:1, which means that it consumes one OOH^•^ and then generates one O₂^•^⁻ to form LO_2_^2−^. In addition, the reaction rate constant of OOH^•^ to LH^•^ (1.4 × 10^9^ L mol^−1^ s^−1^) is tenfold than that of O₂^•^⁻ to LH^•^ (2 × 10^8^ L mol^−1^ s^−1^).^[^ ^40^ ^]^ It suggests that the generated adsorbate OOH• possessed much faster reaction rate to produce the excited state of luminol. Thus, the dominant factor to generate the excited state of luminol is attributed to the adsorbate OOH*.

To demonstrate the high ECL intensity at the edge of Cu─TCNQ, we performed COMSOL to simulate the current density of the Cu─TCNQ/CF electrode by 3D finite element method (details seen in the Supporting Information). A physical field of secondary current distribution was set up to reflect OER current due to the positive relationship between OER current and ECL intensity. If only the macroscopic response is focused on and the pore effect can be ignored, the nonporous electrode model can simplify the calculation. Considering that what we are studying was a macroscopic electrode, we simplify the calculation and ignore the influence of the porosity of the electrode.

**Figure 4:**
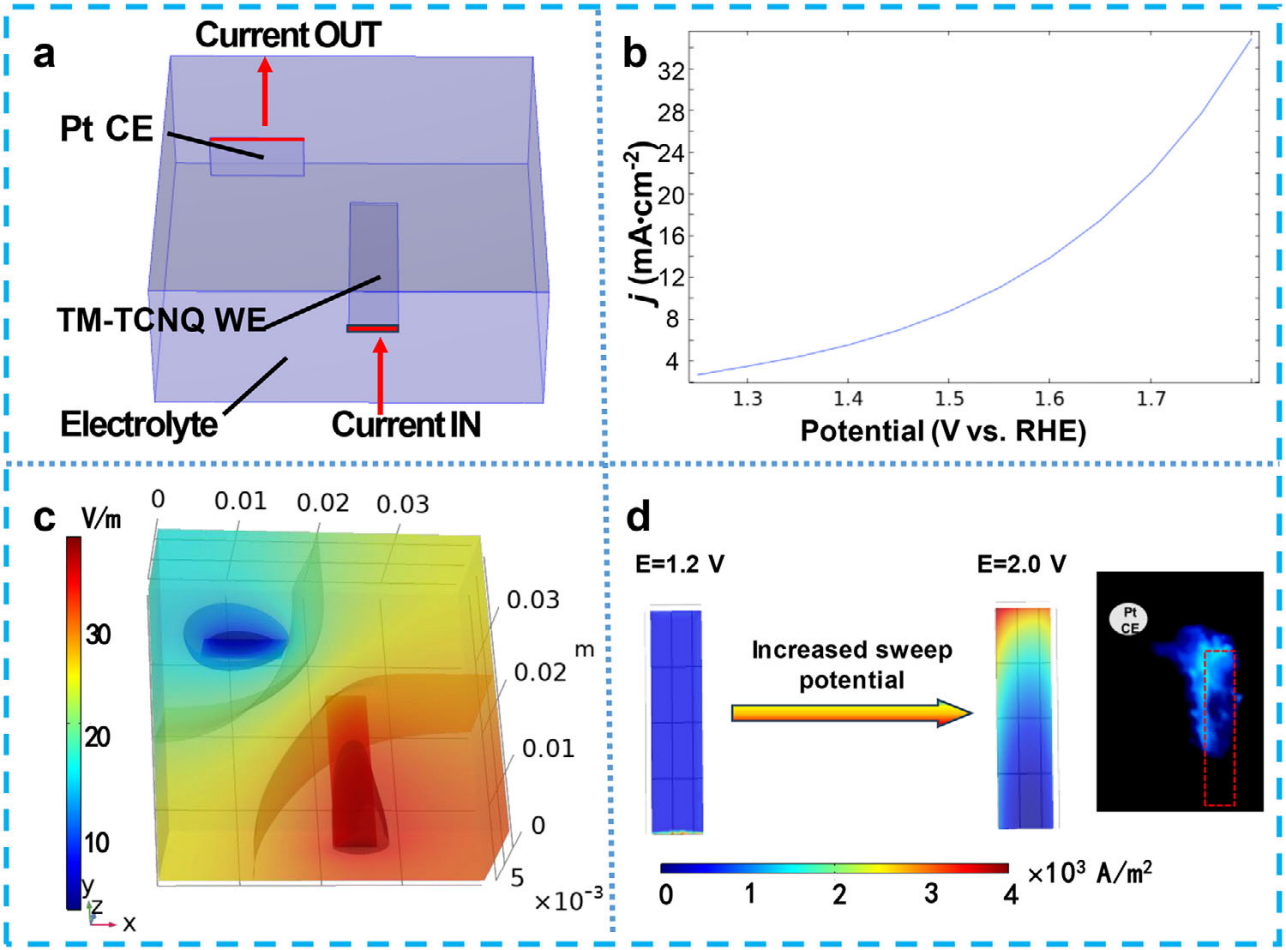
a) Definition of a 3D geometric model. b) Polarization curve from Secondary Current Distribution Multiphysics. c) Electrolyte potential distribution at *E* = 2.0 V. d) Current density distribution images of TM─TCNQ/CF working electrode at different potentials. The inset picture is the ECL photo of the Cu─TCNQ/CF electrode at the constant potential *E* = 2 V, which was shot by a phone in the dark room, and the red frame is the outline of the Cu─TCNQ/CF electrode.

As shown in **Figure** **4**a, we arranged the working electrode and counter electrode according to the actual location. In this simulation, the working electrode Cu─TCNQ and the counter electrode Pt plane were set as the current in and current out, respectively. Figure 4b showed a simulated OER polarization current curve calculated by secondary current distribution in the physical field (*E*_eq_ = 1.23 V). The simulated curve showed a gradually enhanced current density with the voltage positively sweeping, in agreement with the actual OER current curve. The potential distribution in the entire electrolytic cell at *E* = 2.0 V versus RHE was displayed in Figure 4c. At *E* = 2.0 V versus RHE, the OER has been occurring in Cu─TCNQ electrode, and thus, we observed a potential gradient throughout the electrolytic cell from working electrode to counter electrode. In the neutral mass transfer governing equation (Equation (S15), Supporting Information), the density of the equipotential plane represents the electric field gradient ∇*ϕ*_Ɩ_ of migrating mass transfer. As we see, the largest electric field gradient was located in the straight line from the working electrode and the counter electrode. In addition, the simulated OER current density was shown in Figure 4d. Although the Cu─TCNQ/CF working electrode was regarded as an equipotential body with uniform potential distribution, the OER current was stronger at the edge of the electrode when the potential applied exceeded 1.23 V. The current density distribution was also consistent with the ECL intensity distribution in the photo of Figure 4d inset. For example, the OER current at the electrode region close to the counter electrode was stronger than that far away from the counter electrode. It also suggested that the edge maintained rod morphology with good electrical conductivity, whereas the bulk body of the electrode transformed to a more stable flaky morphology MOF (phase II) at high oxidation potential. Because the electric field gradient determined the electromigration mass transfer flux, we observed an interesting phenomenon that the ECL emission layer extended toward the counter electrode (inset in Figure 4d). The coreactant endoperoxide LO_2_^2−^ (Equation (S7), Supporting Information) and electronegative O_2_^•−^ (Equation (S8)) generated in OER process flowed in the direction of electric field gradient, which made the ECL layer thicker and point toward the counter electrode. Therefore, the extended ECL layer exactly reflected the mass transfer induced by the electric field gradient in OER processes.

In addition to Cu─TCNQ, we prepared a series of TM─TCNQ based on Cu─TCNQ as a precursor by cation solution exchange method (Scheme 1). Cu atom as the metal center of TCNQ MOF was converted into a corresponding transition metal atom in the transition metal–organic solvent. As a result, a series of TM─TCNQ MOFs with the same morphology and structure were obtained. In situ cation exchange method solely altered the metal center and thus ensured their turnover frequency as an intrinsic property, excluding the influence of catalytic site number and electroactive area on the characterization of catalyst activity.^[^ ^41^ ^]^ As shown in Figure S11d,h (Supporting Information), SEM elemental mapping images indicated that some Cu centers in Cu─TCNQ have been replaced by Fe and Co elements. Meanwhile, the morphologies of Fe─TCNQ and Co─TCNQ catalysts (Figure S11a,e, Supporting Information) retained nanorod shape after the cation exchange method, the same as the structure of Cu─TCNQ. Figure S12a–c (Supporting Information) showed the fine consistent XPS spectrum of Fe─TCNQ and Co─TCNQ to Cu─TCNQ. In terms of Co─TCNQ, in the Co 2p region (Figure S12d, Supporting Information), the binding energies at 780.6 and 796.5 eV were assigned to Co 2p3/2 and Co 2p1/2, respectively. The binding energies of two shake‐up satellites at 786.8 and 802.7 eV corresponded to Co with oxidation states (Co^2+^). In the Fe 2p region (Figure S12e, Supporting Information), the two major binding energies at 711.2 and 724.5 eV for Fe 2p3/2 and Fe 2p1/2 correspond to Fe^2+^. The XRD pattern (Figure S12f, Supporting Information) also proved that the three TM─TCNQ MOFs possessed the same structure of crystalline.

**Figure 5:**
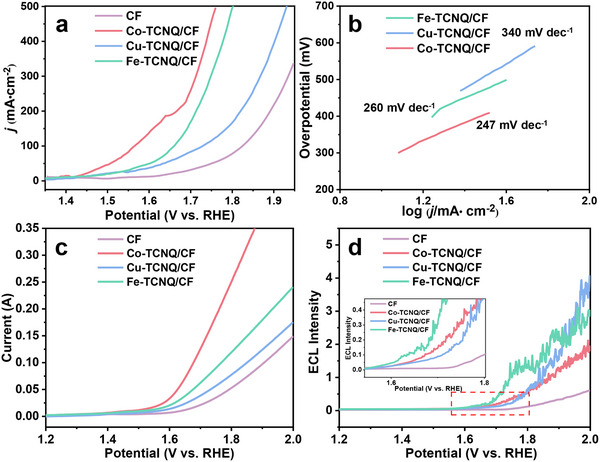
a) LSV curves of bare CF, Cu─TCNQ/CF, Fe─TCNQ/CF, and Co─TCNQ/CF. b) Tafel plots of Cu─TCNQ/CF, Fe─TCNQ/CF, and Co─TCNQ/CF for OER in 1.0 m KOH. Scan rate: 0.002 V s^−1^. c) LSV and d) *I*_ECL_–potential curves of bare CF, Cu─TCNQ/CF, Fe─TCNQ/CF, and Co─TCNQ/CF in 1.0 m KOH and 100 µm L‐012. Scan rate: 0.01 V s^−1^.

Then, we compared the OER performance of these TM─TCNQ by electrochemical descriptors, including overpotential (*η*) at 50 mA cm^−2^ current density and Tafel slope. The LSV polarization current curves (*j*–*V*) and Tafel slope graphs were obtained in 1.0 m KOH electrolyte. As shown in **Figure** **5**a, all TM─TCNQ (Co, Fe, and Cu) showed good OER activity compared with bare CF electrode. When the potential was swept beyond 1.4 V (vs RHE), the OER current significantly rose. The overpotential of Co, Fe, and Cu─TCNQ/CF at the current density of 50 mA cm^−2^ is 270, 370, and 400 mV, respectively. The Tafel slopes of Co, Fe, and Cu─TCNQ/CF were 247, 260, and 340 mV dec^−1^, respectively (Figure 5b). Therefore, the catalytic activity of OER among the three TM─TCNQ MOFs was Co─TCNQ > Fe─TCNQ > Cu─TCNQ.

**Figure 6:**
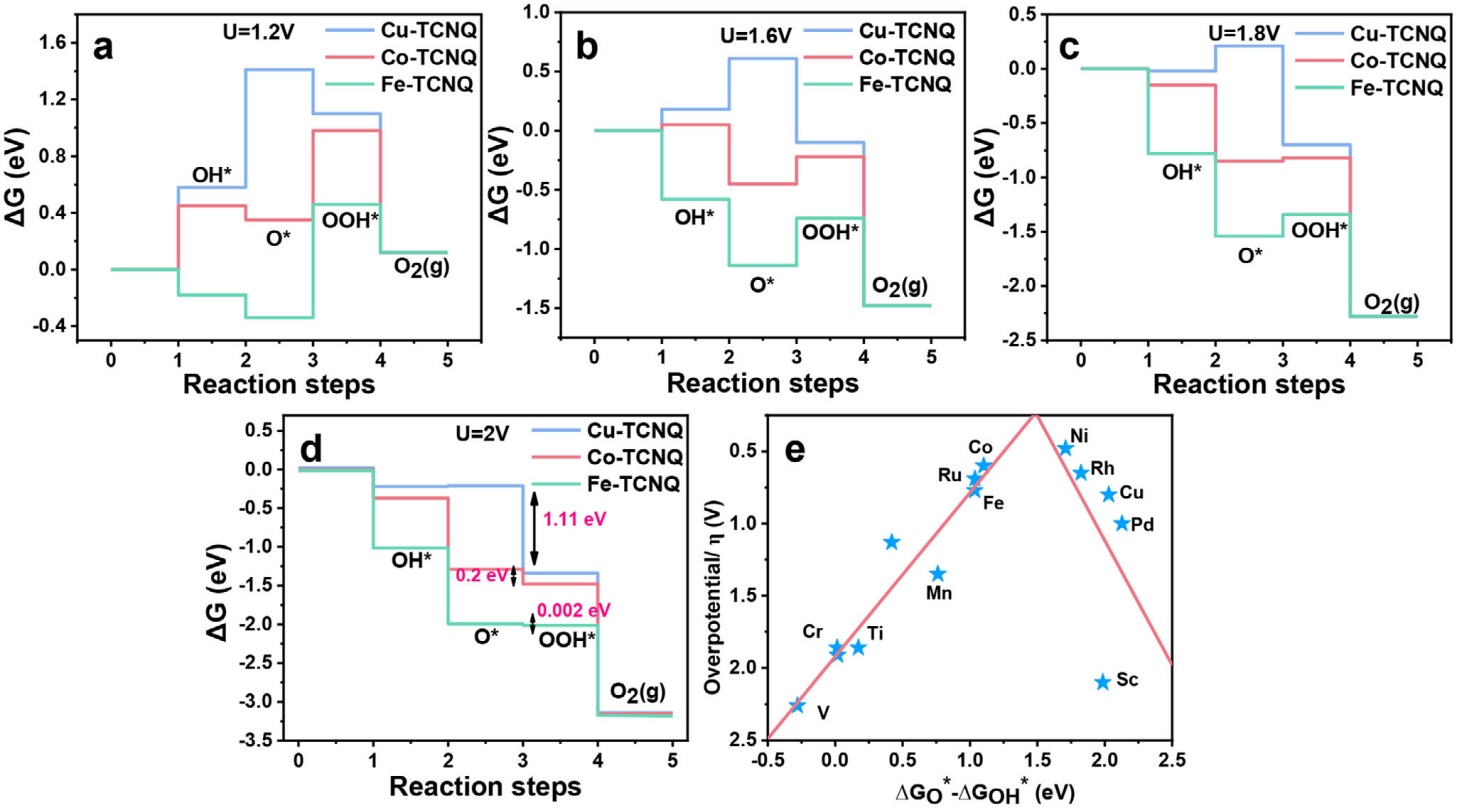
a–d) Gibbs free energy profile for TM─TCNQ catalysts in basic medium (pH = 14) at the different electrode potentials. e) Volcano type plot for TM─TCNQ monolayers with ∆*G*_O*_ − ∆*G*_OH*_ as descriptor.

As shown in Equations (1)–(4), the process of MOF catalyzing OER was divided into four electron transfer reactions. However, these four electron reactions cannot be clearly distinguished one by one by the common electrochemical methods. In other words, the OER kinetics was determined by a certain step among the four electron reactions, but the electrochemical measurements failed to reveal the rate‐determining step. As a complementary technique, the ECL measurements were related to the concentration of OOH* during OER processes at the forward scan. The onset ECL potential of TM─TCNQ (*η*_TM‐ECL_) represented that enough OOH* species in the OER process were generated to enhance the ECL intensity, which directly reflected the kinetic sequence of OOH* generated by different TM─TCNQ. Admittedly, the *η*_TM‐ECL_ of TM─TCNQ/CF was generally larger than the overpotential of OER because OOH* needs to be accumulated until reaching a certain concentration to enhance the ECL signal of L‐012 (Figure 5c,d). Among the three TM─TCNQ, we found the onset ECL potential followed the trend *η*_Fe‐ECL_ < *η*_Co‐ECL_ < *η*_Cu‐ECL_ from the enlarged image of Figure 5d. Interestingly, the sequence of onset ECL potential was inconsistent with the sequence of overpotential *η*_Co_ < *η*_Fe_ < *η*_Cu_ that we got in the electrochemical tests (Figure 5b). Previous reports showed the dynamic and thermodynamic processes during OER of TM─TCNQ by density functional theory (DFT), which calculated the adsorption free energy of each reaction intermediates involved in OER.^[^ ^42^ ^]^ The specific equations to calculate the adsorption free energy of each reaction intermediates on the TM─TCNQ are shown in Equations (S1)–(S8) (Supporting Information). Therefore, we used the DFT data (Table S2, Supporting Information) to interpret the results of electrochemical and ECL measurements. First, the overpotential *η*_Co‐50 mA cm−2_ < *η*_Fe‐50 mA cm−2_ < *η*_Cu‐50 mA cm−2_ obtained by electrochemical test was indeed consistent with the theoretically calculated overpotential *η* (0.60 < 0.77 < 0.88) in DFT.^[^ ^42a^ ^]^ It was because the definition of overpotential, as shown in Equation (S4) (Supporting Information), was determined by the rate‐determining step in the four‐electron transfer. As shown in **Figure** **6**a–d, the TM─TCNQ will undergo a change in the thermodynamic energy level with different potentials applied, and then the specific step of OER will become spontaneous processes when this and the previous energy level were all downhill to <0 eV. For example, with the increase in the applied potential, the thermodynamic energy level for generating OOH* became downhill to <0 eV, and producing OOH* followed the order of Fe─TCNQ > Co─TCNQ > Cu─TCNQ. When the concentration of OOH* was high enough to be responded to L‐012, the onset ECL potential (*η*_TM‐ECL_) appeared. Different from the overpotential *η*_TM‐50 mA cm−2_, *η*_TM‐ECL_ was only determined by ∆*G*_OOH*_ from Equation (S3) (Supporting Information), which represented the Gibbs free energy from the start of OER to the production of OOH*. Based on the bridge equation (∆*G* = −*zFE*) linking electrochemistry and thermodynamics, we believed that *η*_TM‐ECL_ was directly related to free energy. It was also reasonable to correlate *η*_TM‐ECL_ obtained by the ECL–potential curves with the free energy (∆*G*_OOH*_). The order (*η*_Fe‐ECL_ < *η*_Co‐ECL_ < *η*_Cu‐ECL_) obtained by ECL measurements was consistent with the DFT simulation where ∆*G*_OOH*_ of the three TM─TCNQ was calculated according to Equation (S3) (Supporting Information) (∆*G*_Fe_: 4.06 (eV) < ∆*G*_Co_: 4.58 (eV) < ∆*G*_Cu_: 4.70 (eV)). Therefore, we provided experimental evidence for this DFT result for the first time.

However, when the scanning potential reached *E* = 2 V versus RHE, the tendency of existence and accumulation as the form of OOH* on TM─TCNQ were determined by the energy barrier between adjacent intermediates of O* and OOH*. As shown in Figure 6d, the energy level difference of Cu─TCNQ was the largest at downhill (∆*G* = −1.11 eV, calculated according to Equation (S7), Supporting Information) among the three TM─TCNQ, while the energy level differences of both Co─TCNQ and Fe─TCNQ were close to 0 eV. Therefore, the generated OOH* during OER was more easily to be accumulated on Cu─TCNQ and showed the strongest intensity compared with Co─TCNQ and Fe─TCNQ in Figure 5d. It was also verified by recording the ECL intensity trajectories of the three TM─TCNQ under a constant potential (*E* = 2 V vs RHE) (Figure S13a,b, Supporting Information). Initially, the current–time curves of TM─TCNQ gradually declined under the constant voltage as described with the Cottrell equation. However, all the ECL curves showed a gradual increase at first until reaching the peaks afterward. The ECL intensity peaks at *E* = 2 V of the three TM─TCNQ showed the same sequence under CV scan (Figure S13d, Supporting Information).

As we know, the OER volcano‐type plot (Figure 6e) was often used to find suitable catalysts and predict the performance of unreported catalysts. The volcano‐type plot of TM─TCNQ OER was drawn with ∆*G*_O*_ − ∆*G*_OH*_ as a descriptor by the data of DFT.^[^ ^43^ ^]^ According to the volcano map, the catalysts on the left side of the vertices have stronger bonding to OH*, making it difficult to form OOH*. By contrast, the catalysts on the right side easily proceed to become OOH*.^[^ ^44^ ^]^ Because Cu─TCNQ was located on the right side of the vertices, Cu─TCNQ was the easiest to accumulate OOH* among the three TM─TCNQ. Nevertheless, we also found that the ECL intensities of Co─TCNQ and Fe─TCNQ at *E* = 2 V do not reflect their energy barrier from the DFT simulation. We thought that it was attributed to the insufficient cation exchange in the synthesis process of Fe─TCNQ. To prove this point, we used ICP‐AES to quantify the metal elements in the three TM─TCNQ (Table S1, Supporting Information). For Fe─TCNQ, the proportion of the Fe element in Fe─TCNQ is 30% of that of the Cu element. Therefore, the insufficient cation exchange made the ECL intensity of Fe─TCNQ close to that of Cu─TCNQ at *E* = 2 V. Despite the small percentage, Fe─TCNQ still advanced the onset ECL potential and decreased the ECL intensity compared with pure Cu─TCNQ.

To screen good OER catalysts, it is necessary to find the rate‐determining step among the OER four‐electron transfer. For most OER catalysts, the rate‐determining step was the O* (∆*G*_2_) and OOH* (∆*G*_3_) reaction steps.^[^ ^45^ ^]^ According to the ECL–potential curve, we distinguished the rate‐determining step between ∆*G*_2_ and ∆*G*_3_ through the onset ECL potential of TM─TCNQ (*η*_TM‐ECL_, determined by ∆*G*_OOH_) and the accumulated ECL intensity at constant potential *E* = 2 V (*I*_TM‐ECL_, determined by ∆*G*_3_). Taking Cu─TCNQ as an example, the onset ECL potential of Cu─TCNQ (*η*_Cu‐ECL_) was the largest among the three TM─TCNQ. It suggested that Cu─TCNQ was the slowest and the last to produce OOH* at kinetics in OER. However, the accumulated ECL intensity of Cu─TCNQ (*I*_Cu‐ECL_) was higher than the others at *E* = 2 V. It indicated that the OOH* tended to be accumulated easily on Cu─TCNQ at thermodynamic. Thus, for Cu─TCNQ, the rate‐determining step was ∆*G*_2_. For Co─TCNQ, *η*_Co‐ECL_ was just smaller than *η*_Cu‐ECL,_ but *I*_Co‐ECL_ was the lowest among the three, suggesting the weak ability to accumulate OOH*. Thus, ∆*G*_3_ was the RDS for Co─TCNQ. Finally, for Fe─TCNQ, the *η*_Fe‐ECL_ was the smallest but harder to generate OOH* compared with Cu─TCNQ, so the most probability of the RDS was ∆*G*_3_.

The adsorption energies of the OER intermediates including OH*, OOH*, and O* species that participate in the AEM are linearly correlated. In particular, both OOH* and OH* bind with the catalyst surface through an oxygen atom via a single bond, the binding energies of OH* and OOH* are tightly linked with a constant difference (Δ*G*_OOH*_ − Δ*G*_OH*_) of 3.2 ± 0.2 eV for either metals or oxide surfaces, regardless of the binding site (Figure S14, Supporting Information). Hence, the value of Δ*G*_OOH*_ can be directly obtained from the calculated Δ*G*_OH*_ and vice versa, reducing the required computational cost to assess the activity of a given catalyst. Similarly, we try to build a quantitative relationship between ECL overpotential (*η*_TM‐ECL_, Δ*G*_OOH*_) and OER thermodynamic energy by the scaling relationship between the adsorption free energies of adsorbates (Equations (S9) and (S10), Supporting Information). We choose *η*_TM‐ECL_ data at the 0.1 a.u. (ECL intensity) from Figure 5d, normalizing them, and then converting them proportionally to ∆*G*_OOH*_. The result in Table S3 (Supporting Information) is calculated by Equations (S9) and (S10) (Supporting Information) and is consistent with the conclusion of the rate‐determining step. Therefore, the clarification in the RDS by ECL measurements was in good agreement with the theoretical results calculated by DFT. Knowing the OER determination step of MOF materials was of great significance in optimizing the catalytic activity of OER. Researchers will further improve the OER activity by adjusting the electronic structure,^[^ ^7^ ^]^ coordinating axial ligands,^[^ ^46^ ^]^ and applying external strain.^[^ ^47^ ^]^ For example, when Fe as the central element in SAC 2D MOF was grafted with CN^−^ (cyano group) axial ligand, the energy level of ∆*G*_3_ was greatly optimized, and thus OER activity was significantly enhanced.^[^ ^48^ ^]^ Therefore, it is of practical significance to perceive the rate‐determining step—a part of OER to master the whole catalytic process.

## 3 Conclusion

In summary, we synthesized Cu─TCNQ MOF with two different phases (phase I and phase II) as an effective OER model catalyst. The ECL probe L‐012 specifically was oxidized by OOH* and thus emit ECL in situ. Thanks to the spatial and temporal resolution of ECL microscopy, we visualized and differentiated OER activity by the evolution of the ECL signal. The ECL images demonstrated that the edge of Cu─TCNQ showed better OER activity due to the better electrical conductivity of rod‐shaped phase I. Interestingly, we discovered the local ECL enhancement at the interface of oxygen microbubbles generated under a high anodic voltage. This phenomenon offered visualized evidence that the ECL localizes on oxygen microbubbles during OER for the first time. The ECL distribution on the electrode surface was verified by COMSOL simulation, which explained the stronger and extended ECL layer toward the counter electrode. To expand this ECL method for the evaluation of OER, we compared OER performance and ECL–potential curves of three kinds of TM─TCNQ. The ECL curves enabled us to distinguish the rate‐determining step of OER for the three TM─TCNQ, where the generation and accumulation of OOH* were determined by *η*_TM‐ECL_ and *I*_TM‐ECL_. Because the ECL evolution was directly related to the OOH* generation and transformation during four‐electron OER, we expected this ECL assessment to provide an alternative approach to analyze OER heterogeneity and select suitable catalysts with high throughput.

## Conflict of Interest

The authors declare no conflict of interest.

## Supporting information



Supporting Information

Supporting Information

## Data Availability

The data that support the findings of this study are available from the corresponding author upon reasonable request.
