# Supplementary material for: Electrochemiluminescence Discloses Active Intermediates in Oxygen Evolution Reactions of Metal–Organic Framework
Source: Adv Sci (Weinh). 2025 Jun 20;12(34):e06309. doi: 10.1002/advs.202506309 (PMC13001627; doi:10.1002/advs.202506309)
Supplement: Supplementary file 1 — Supporting Information [file ADVS-12-e06309-s002.pdf]

Supporting Information

Electrochemiluminescence Discloses Active Intermediates in Oxygen Evolution Reactions of Metal-Organic Framework

Xuan Chen[a], Bing-Xin Sun[a], Wei Xu[a], Chen-Hui Yin[a], Shi-Yi Zhou[a], Yi-Han Qian[a], Lu-Nan Zhang[a], Qin Xu[a]*, Huan Pang[a]* and Cheng Ma[a]*

[a]School of Chemistry and Chemical Engineering, Yangzhou University, Yangzhou, 225002, China

*Correspondence email: xuqin@yzu.edu.cn; panghuan@yzu.edu.cn chengma@nju.edu.cn;

Table of Contents

Experimental Section 3
Synthesis of TM-TCNQ 3
Characterization of Cu-TCNQ 4
Electrochemistry and ECL measurement of Cu-TCNQ 5
ECL images of Cu-TCNQ 7
Characterization of TM-TCNQ 9
Electrochemistry and ECL measurement of TM-TCNQ 11
Supporting Data 12
Reference 16
Author Contributions 16

Experimental Section

Materials and Reagents

Potassium hydroxide (KOH), methanol, ethyl alcohol, acetonitrile, cobaltous nitrate hexahydrate ($Co(NO_3)_2 \cdot 6H_2O$), benzoquinone, hydrogen peroxide (30%) and thiourea were purchased from Aladdin. 7,7,8,8-tetracyanoquinodimethane (TCNQ) and ferrous sulfate ($FeSO_4$) are from Adamas-beta. L-012 was got from Wako Co., Ltd. (Japan). All above reagents were of analytical grade.

Apparatus

ITO glass (surface resistivity <10 Ω/square, thickness 1800 Å) was from Zhongjingkeyi Technology Co., Ltd and Copper foam (0.8 mm × 100 mm × 100 mm) was obtained from Ke Sheng He Co., Ltd. (Su Zhou China) as WE. A platinum sheet (10 mm × 10 mm) as the counter electrode, and an Ag/AgCl (saturated KCl) as the reference electrode. CHI 660D electrochemical workstation (CH Instruments Co., China) and the photomultiplier tube (PMT) for capturing electrochemical signals and ECL emission, respectively. A CMOS camera (TELEDYNE PHOTOMETRICS Prime 95B, China) was utilized to collect ECL images from objective. The ECL images were collected in the dark room. The SEM and element mapping images were observed with a Zeiss_Supra55 field-emission scanning electron microscope. D8 ADVANCE multifunction X-ray diffractometer and ESCALAB 250Xi were used to record powder X-ray diffraction patterns (PXRD) and XPS spectra, respectively.

Synthesis of TM-TCNQ

Preparation of Cu-TCNQ/CF

A piece of copper foam (CF) (5 mm × 40 mm) was carefully pre-treated by three

steps with hydrochloric acid, ethanol and deionized water and every step was for about 10 minutes to remove the surface oxide and impurities. The pre-treated CF was dried in vacuum oven at 60ºC and then immersed in 6 mL acetonitrile which including 8.58 mg TCNQ at 60ºC for 2.5 h. The solution turned green in color, indicating the formation of Cu-TCNQ. After that we immersed the Cu-TCNQ (phase I) in pure acetonitrile for 0.5 h until the solution turned yellow representing the formation of Cu-TCNQ (phase I and phase II). The sample was rinsed several times with deionized water and dried prior to characterization.

Preparation of Fe-TCNQ /CF and Co-TCNQ/CF

Fe-TCNQ and Co-TCNQ nanorods on copper foam were synthesized by cationic exchange from Cu-TCNQ. The Cu-TCNQ/CF (phase I and phase II) precursors were immersed in 20 mL methanol containing 20 mg $FeSO_4$ (slightly soluble) at 60ºC for 4 h to get Fe-TCNQ /CF or in 10 mg mL^{-1} $Co(NO_3)_2{\cdot}6H_2O$/ methanol solution at 60ºC for 4 h to get Co-TCNQ/CF. The sample was rinsed several times with deionized water and dried.

Characterization of Cu-TCNQ

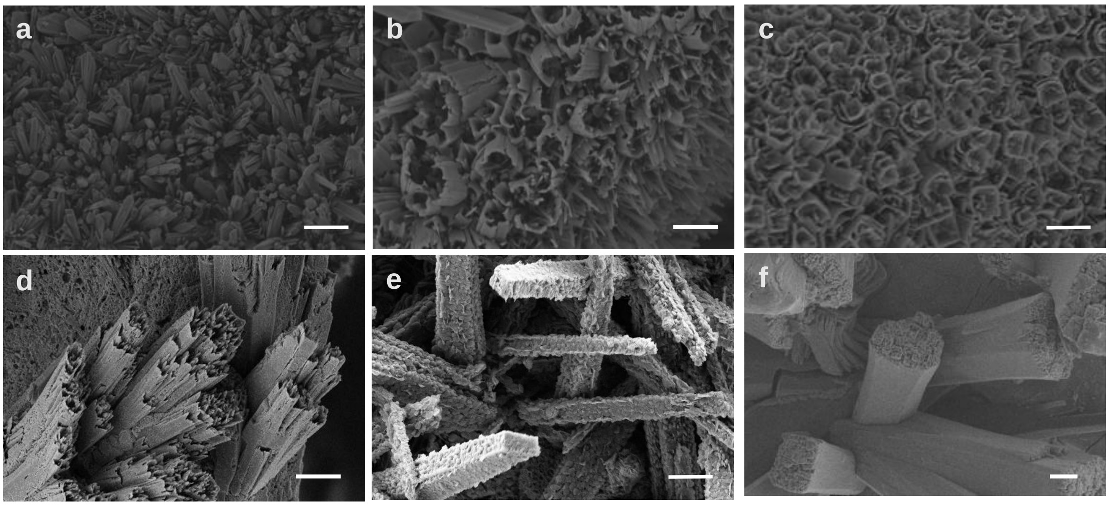

Figure S1. The growth of Cu-TCNQ/CF from phase I to phase II. SEM images of synthesis time for (a) 1 min, (b) 5 min, (c) 15 min, (d) 0.5 h, (e) 3 h, (f) 24 h of Cu-TCNQ at 60ºC. Scale bar: 2 μm.

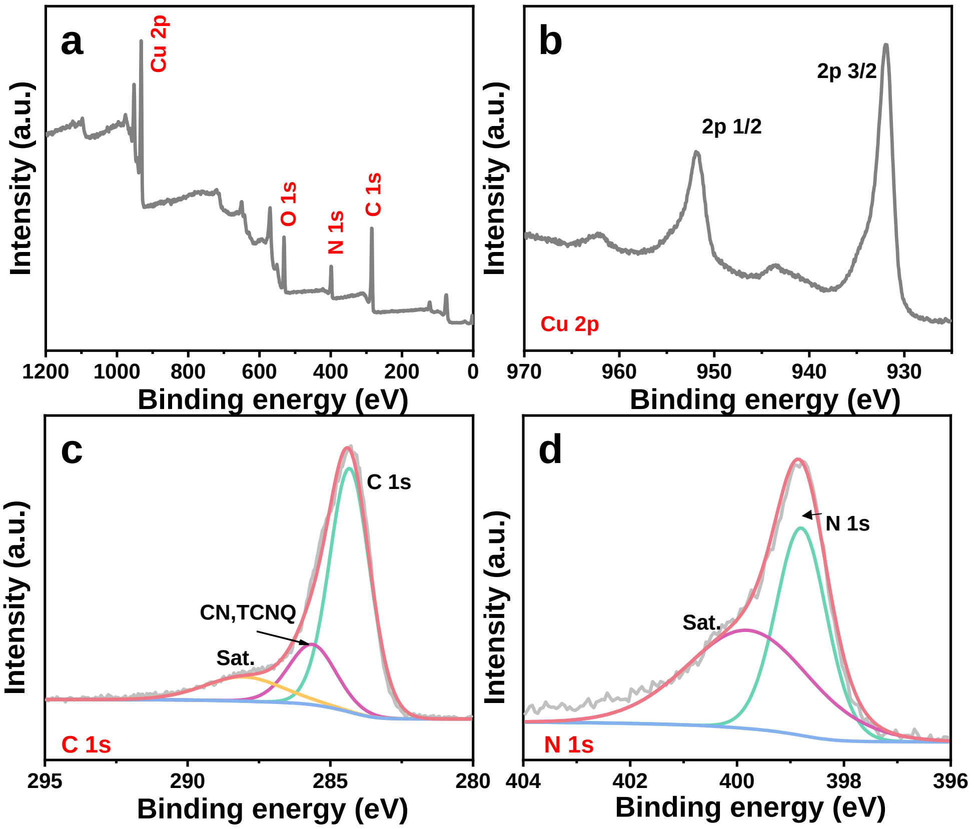

Figure S2. (a) XPS spectra for Cu-TCNQ/CF. (b) Cu 2p, (c) C 1s and (d) N 1s regions for Cu-TCNQ/CF.

Electrochemistry and ECL measurement of Cu-TCNQ

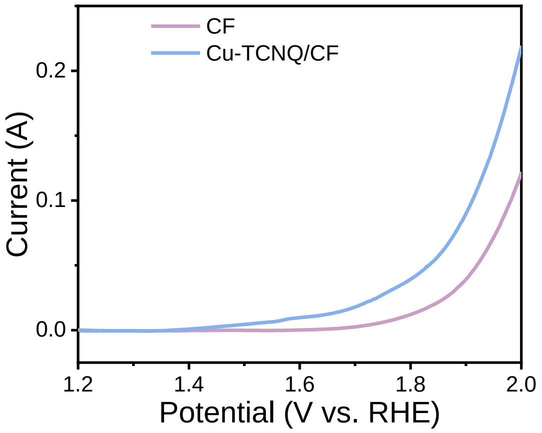

Figure S3. LSV curves of CF and Cu-TCNQ/CF in 1.0 M KOH. Scan rate: 0.005 V/s.

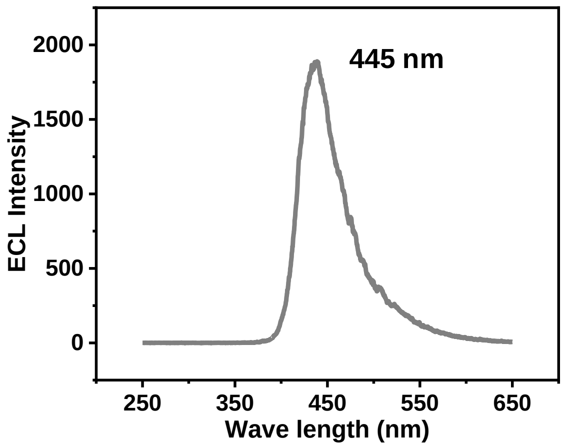

Figure S4. The ECL spectrum during Cu-TCNQ/CF catalyzing OER with L-012 probe addition.

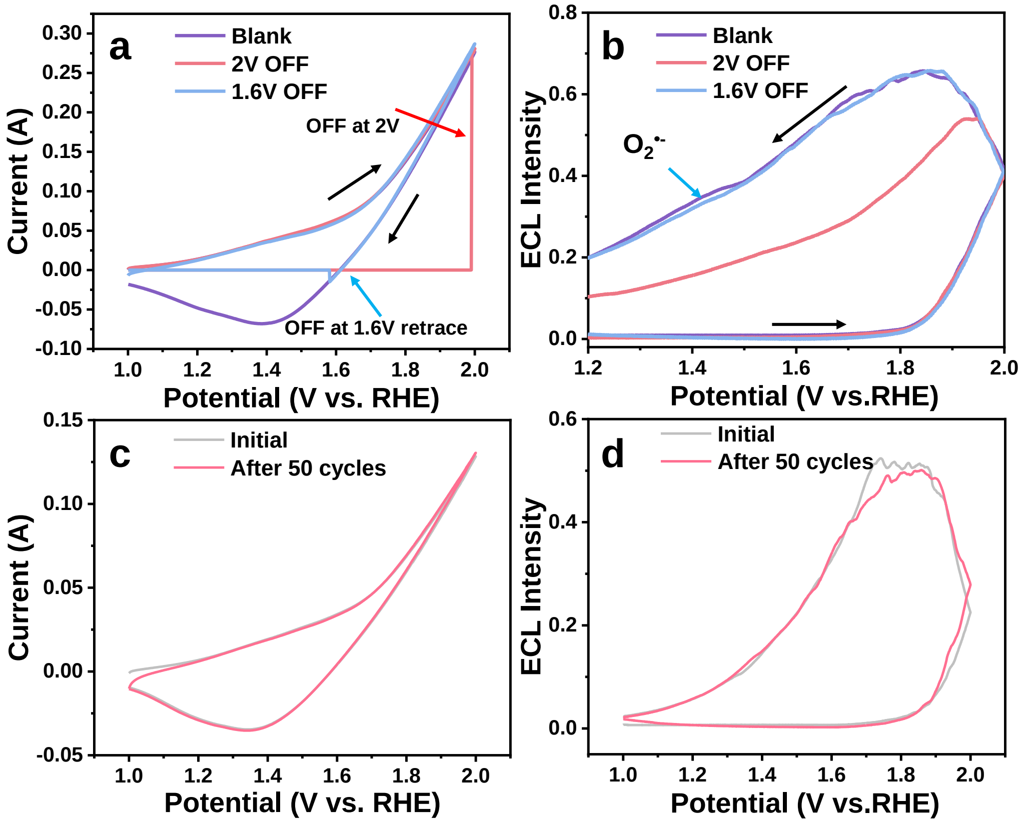

Figure S5. (a) CV and (b) ECL-potential curves for Cu-TCNQ/CF when potential was halted at different potential. (c) CV curves for Cu-TCNQ/CF before and after 50 cyclic sweeping in 1.0 M KOH. (d) I_{ECL}–potential curves before and after 50 cyclic sweeping in the electrolyte containing 1.0 M KOH and 100 μM L-012. Scan rate: 0.1 V/s.

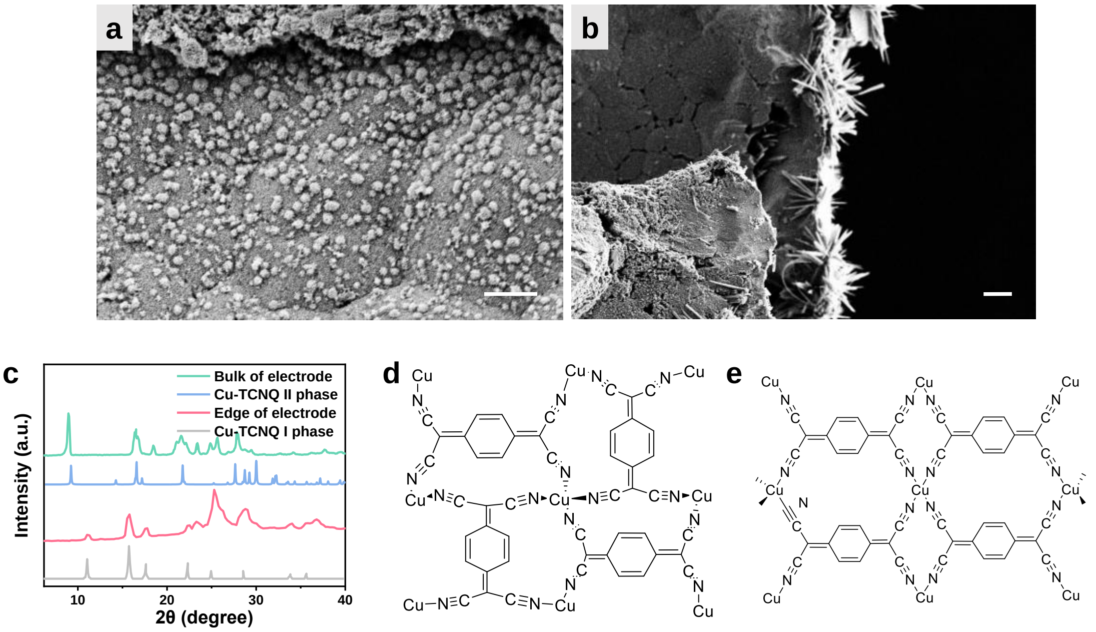

Figure S6. SEM images of Cu-TCNQ/CF (a) bulk and (b) edge. Scale bar: 5 μm. (c) XRD pattern of the nanoparticle from the bulk and edge of electrode, simulated pattern to I and II phase of Cu-TCNQ. Schematic drawings of the Cu-TCNQ (d) phase I and (e) phase II.

ECL images of Cu-TCNQ

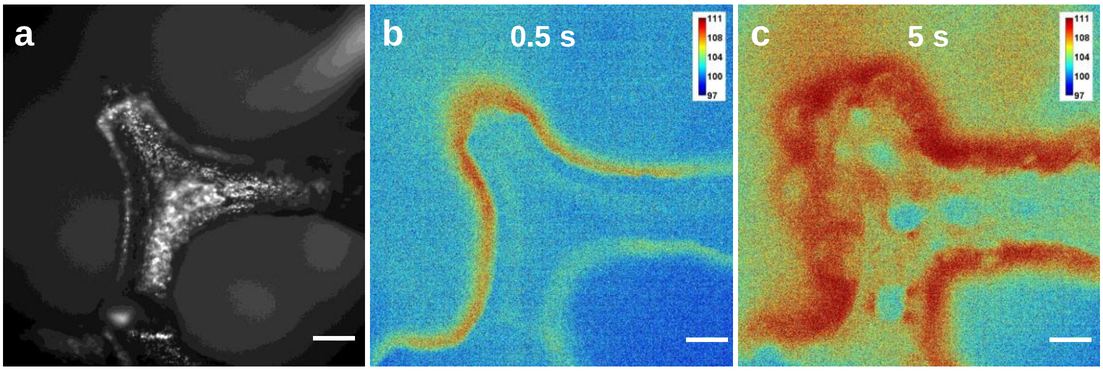

Figure S7. (a) Bright field image, (b) ECL images of Cu-TCNQ/CF electrode mesh and (c) bubble on it versus different reaction times holding at constant potential E= 2 V in the electrolyte with 1 M KOH and 1 mM L-012. Exposure time 200 ms. Scale bar: 20 μm.

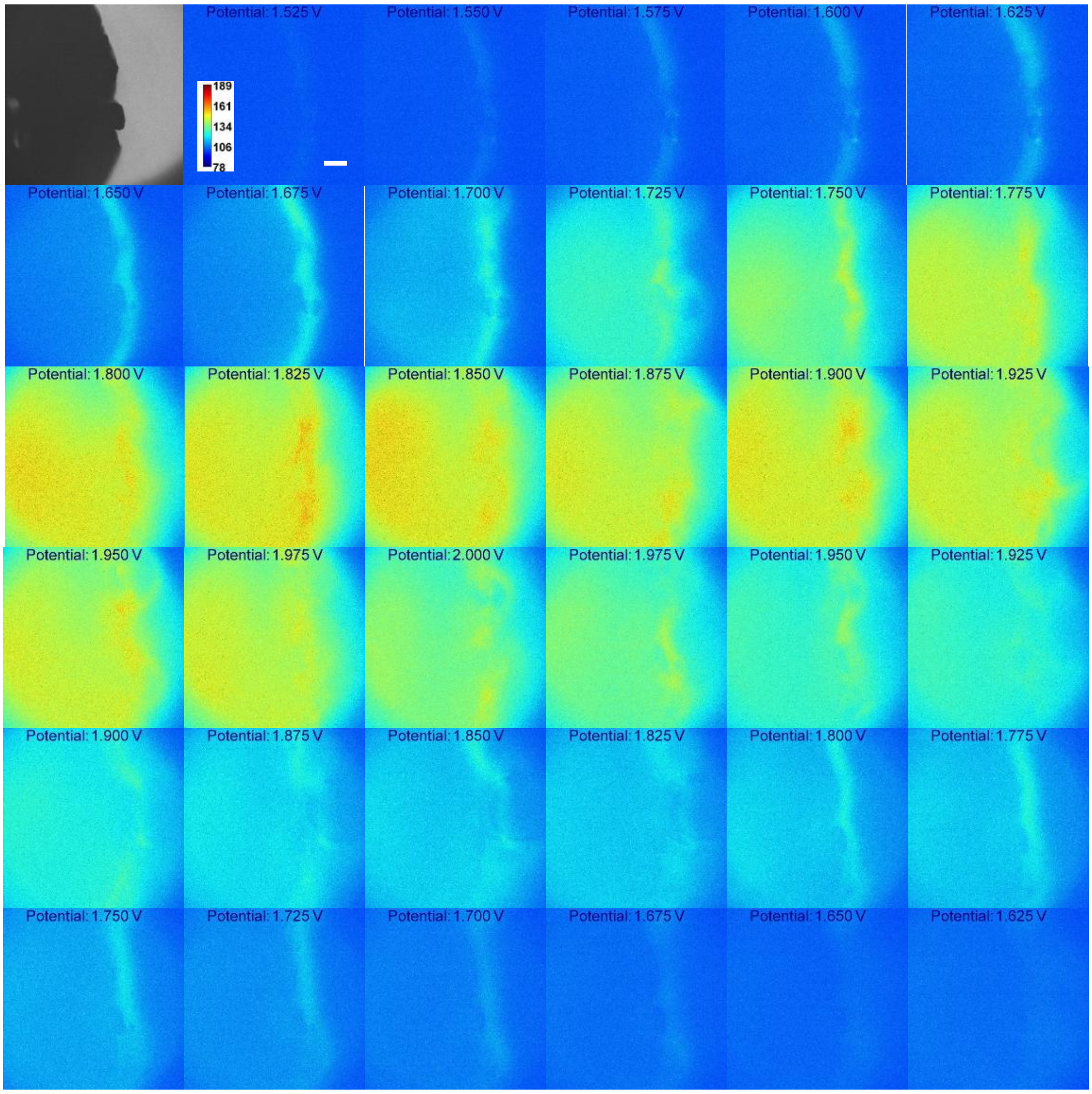

Figure S8. Successive ECL snapshots of Cu-TCNQ/CF catalyzing OER with a cyclic voltammetry scan. Scan range from 1 V to 2 V vs.RHE. Scan rate: 0.1 V/s. Electrolyte is 1 M KOH and 1 mM L-012. Exposure time 100 ms. Scale bar: 20 μm.

ECL images of micro bubble

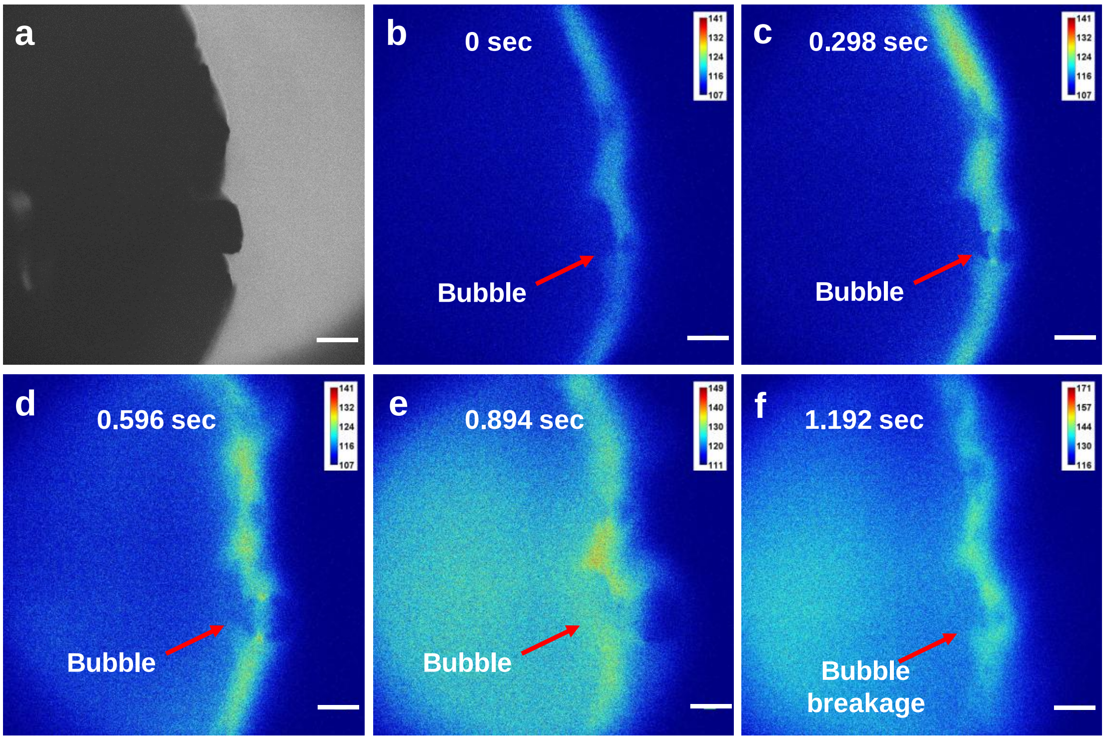

Figure S9. (a) Bright field image of Cu-TCNQ/CF electrode and (b-f) ECL image recorded the generation and development of a micro bubble during OER CV scan (1.0 - 2.0 V) in 1 M KOH and 1 mM L-012 electrolyte. Exposure time: 100 ms. Scale bar: 20 μm.

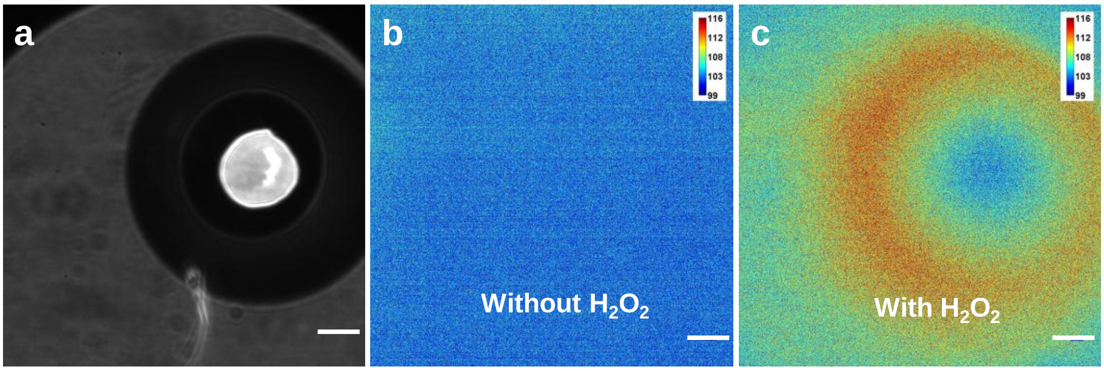

Figure S10. (a) Bright field image of a CO_2 micro bubble on the ITO electrode. ECL images of the bubble were recorded at a constant potential (2.0 V) without (b) or with (c) 1 mM H_2O_2 addition in 0.1 M PBS (pH=7.4) and 1 mM L-012 electrolyte. Exposure time: 1000 ms. Scale bar: 20 μm.

Characterization of TM-TCNQ

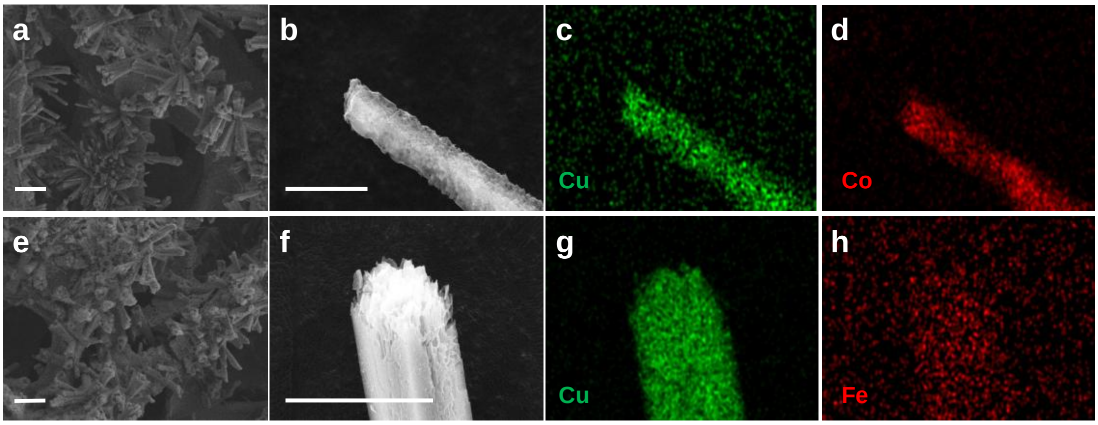

Figure S11. SEM images of (a) Co-TCNQ/CF and (e) Fe-TCNQ/CF. Scale bar: 10 μm, (b) and (f) their single rod, respectively. (c, d, g, h) Elemental mapping images of Cu, Fe, and Co elements for Fe-TCNQ and Co-TCNQ nanorod.

Table S1. ICP-AES data for different samples scratched down from TM-TCNQ/CF.

Sample	Cu (mg/L)	Fe (mg/L)	Co (mg/L)
**Cu-TCNQ**	13.35	--	--
**Fe-TCNQ**	9.72	3.89	--
**Co-TCNQ**	0.97	--	12.41

The Cu-TCNQ catalyst mass loading was ~14 mg/cm² on Cu-TCNQ/CF. The Fe-TCNQ catalyst was consistent at 4.5 mg/cm² Fe-TCNQ and 11.3 mg/cm² Cu-TCNQ. The Co-TCNQ catalyst was consistent at 13.8 mg/cm² Co-TCNQ and 1.1mg/cm² Cu-TCNQ. The specific steps are as follows:

Loading amount (mg/cm^2) = $(\rho \times V \times M_{TM\text{-}TCNQ}/M_{TM})/A$

ρ: Mass concentration measured by ICP-AES (mg/L)

V: Volume of nitric acid

$M_{TM\text{-}TCNQ}$: Molecular weight of TM-TCNQ

M_{TM}: Molecular weight of TM

A: The effective area of the material substate

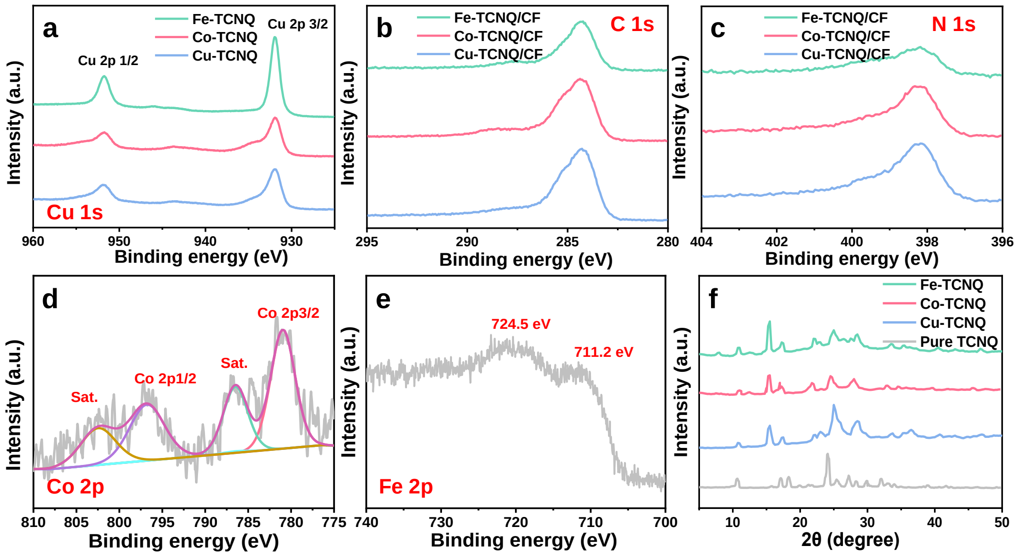

Figure S12. (a) XPS spectra for TM-TCNQ/CF. (b) C 1s, (c) N 1s, and (d) Co 2p regions for Co-TCNQ/CF, (e) Fe 2p regions for Fe-TCNQ/CF. (f) XRD pattern of TM-TCNQ/CF.

Electrochemistry and ECL measurement of TM-TCNQ

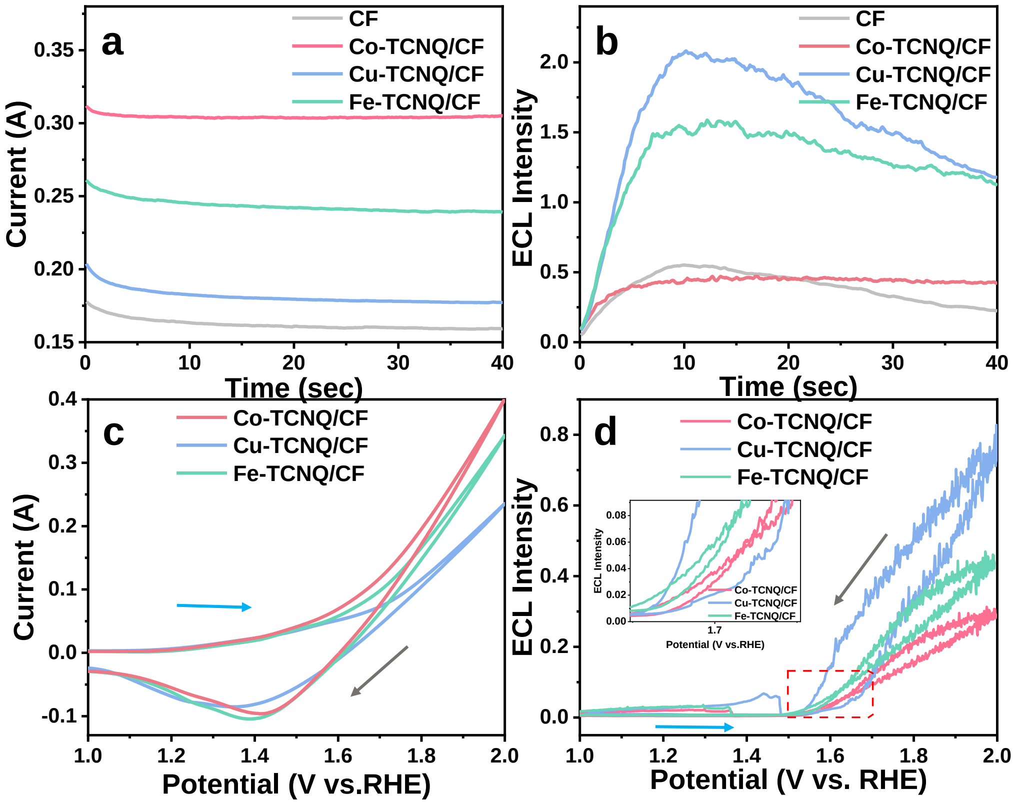

Figure S13. Current (a) and ECL intensity versus time (b) curves holding at the constant potential E = 2 V; (c) CV and I_{ECL}–potential curves (d) of CF and TM-TCNQ/CF in the electrolyte with1.0 M KOH and 100 μM L-012. Scan rate: 0.005 V.

Supporting Data

Electrochemical measurements and ECL tests for TM-TCNQ/CF

Electrochemical measurements were performed with a CHI 660D electrochemical analyzer in a conventional three-electrode system. TM-TCNQ/CF, Ag/AgCl, and platinum plate were used as working, reference, and counter electrodes, respectively. The current densities were calculated concerning the geometrical area of the electrodes (0.5 cm × 0.5 cm). The reference electrode was calibrated to the reversible hydrogen electrode (RHE) scale in just OER electrochemical measurements using the following equation[1]:

E (RHE) = E(Ag/AgCl) + (0.059 pH + 0.198) V

Overpotentials (η) were calculated by the following equation:

η = E (RHE) – 1.23 V

Polarization curves were obtained using linear sweep voltammetry with a scan rate of 2 mV s^{-1} in 1.0 M KOH electrolyte. All experiments were carried out at room temperature (298 K).

Tafel plots calculation: The Tafel plots are employed to evaluate the OER catalytic kinetics and fitted with the following equation:

η = b log j + a

Where $\boldsymbol{j}$ is the current density and $\boldsymbol{b}$ is the Tafel slope.

DFT calculation

DFT was used to calculate the adsorption free energy of each reaction intermediates on the TM-TCNQ monolayer[2].

$$\Delta G_{OH*} = E_{OH*} - E^{*} + (\Delta ZPE - T\Delta S)_{OH*} - (G_{H_2O} - \frac{1}{2}G_{H_2}) \quad \text{(S1)}$$

$$\Delta G_{O*} = E_{O*} - E^{*} + (\Delta ZPE - T\Delta S)_{O*} - (G_{H_2O} - G_{H_2}) \quad \text{(S2)}$$

$$\Delta G_{OOH*} = E_{OOH*} - E^{*} + (\Delta ZPE - T\Delta S)_{OOH*} - (2G_{H_2O} - \frac{3}{2}G_{H_2}) \quad \text{(S3)}$$

$$\eta^{OER} = \frac{Max\{\Delta G_1, \Delta G_2, \Delta G_3, \Delta G_4\}}{e} - 1.23 \quad \text{(S4)}$$

Where, $\Delta G_1 = \Delta G_{OH*}$ (S5)

$$\Delta G_2 = \Delta G_{O*} - \Delta G_{OH*} \quad \text{(S6)}$$

$$\Delta G_3 = \Delta G_{OOH*} - \Delta G_{O*} \quad \text{(S7)}$$

$$\Delta G_4 = 4.92\ eV - \Delta G_{OOH*} \quad \text{(S8)}$$

Table S2. The ΔG of various reaction intermediates in OER process and calculated overpotential (η) for different TM-TCNQ monolayers[2-3].

Catalyst surface	ΔG_{OH*} (eV)	ΔG_{O*} (eV)	ΔG_{OOH*} (eV)	ΔG_1 (eV)	ΔG_2 (eV)	ΔG_3 (eV)	ΔG_4 (eV)	η (V)
**Sc-TCNQ**	-1.63	0.35	1.58	-1.63	1.98	1.22	3.33	2.10
**Ti-TCNQ**	-1.32	-1.15	1.94	-1.32	0.17	3.09	2.97	1.86
**V-TCNQ**	-0.48	-0.76	2.73	-0.47	-0.28	3.49	2.18	2.26
**Cr-TCNQ**	0.58	0.60	3.74	0.58	0.02	3.15	1.17	1.91
**Mn-TCNQ**	0.80	1.56	4.14	0.80	0.76	2.58	0.78	1.35
**Fe-TCNQ**	1.02	2.06	4.06	1.02	1.04	2.00	0.86	0.77
**Co-TCNQ**	1.65	2.75	4.58	1.65	1.10	1.83	0.34	0.60
**Ni-TCNQ**	1.70	3.41	4.75	1.70	1.71	1.34	0.17	0.48
**Cu-TCNQ**	1.78	3.81	4.70	1.78	2.03	0.89	0.22	0.80
**Ru-TCNQ**	0.71	1.75	3.67	0.71	1.04	1.92	1.25	0.69
**Rh-TCNQ**	1.89	3.71	4.78	1.89	1.82	1.07	0.14	0.65
**Pd-TCNQ**	2.28	4.41	4.89	2.28	2.13	0.48	0.03	1.00

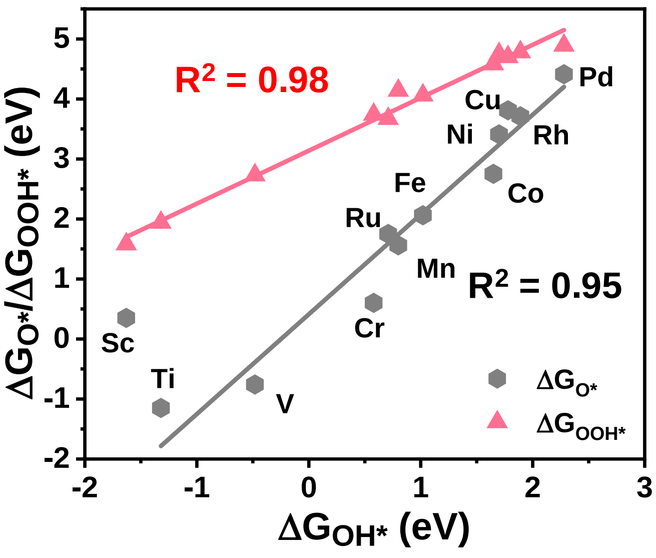

Figure S14. Scaling relationship between the adsorption free energies (ΔG_{OH*} vs. $\Delta G_{OOH*}/\Delta G_{O*}$) of adsorbates on various TM-TCNQ.

Two perfect linear relationships have been observed between ΔG_{OH*} vs ΔG_{O*} and between ΔG_{OH*} vs ΔG_{OOH*} with the fitting equations as follows:

$$\Delta G_{OOH*} = 3.13 + 0.91 \times \Delta G_{OH*} \quad \text{(S9)}$$

$$\Delta G_{O*} = 0.29 + 1.73 \times \Delta G_{OH*} \quad \text{(S10)}$$

Table S3. The ΔG of various reaction intermediates in OER process and calculated from ECL overpotential ($\eta_{TM\text{-}ECL}$) for different TM-TCNQ catalyst.

TM-TCNQ	$\eta_{TM\text{-}ECL}$ (V)	ΔG_{OH*} (eV)	ΔG_{O*} (eV)	ΔG_{OOH*} (eV)	ΔG_2 (eV)	ΔG_3 (eV)	rate-determining step
**Fe-TCNQ**	**0.40 (0.85)**	**1.16**	**2.29**	**4.18 (0.85)**	**1.13**	**1.89**	**Step 3**
**Co-TCNQ**	**0.44 (0.93)**	**1.59**	**3.04**	**4.58 (0.93)**	**1.45**	**1.54**	**Step 3**
**Cu-TCNQ**	**0.47 (1.0)**	**1.97**	**3.69**	**4.92 (1.0)**	**1.72**	**1.23**	**Step 2**

The data in parentheses (0.85, 0.93, 1.0) were obtained from $\eta_{TM\text{-}ECL}$ data at the 0.1 a.u. (ECL intensity) in Figure 5d, and then normalize them. The data in Table S3 is calculated by equation S9 and S10.

COMSOL simulation of Cu-TCNQ/CF macro electrode

COMSOL Multiphysics (Version 5.6) was used for digital simulations[4].

Table S4. Parameter

Name	Value	Description
**Eeq_c**	0 [V]	Cathode equilibrium potential
**Eeq_a**	1.23 [V]	Anode equilibrium potential
**E_cell**	1.5 [V]	Cell potential
**i0_c**	1030 [mA/m^2]	Cathode exchange current density
**Ac**	-149 [mV]	Cathode Tafel coefficient
**i0 _a**	2.56 [mA/m^2]	Anode exchange current density
**Aa**	350 [mV]	Anode Tafel coefficient
**T**	298[K]	Temperture

The governing equations of our finite element model are given by the following relations:

$\nabla \cdot I_l = Q_l$ with $I_l = -\sigma_l \nabla \varphi_l$ (S11)

$\nabla \cdot I_s = Q_s$ with $I_s = -\sigma_s \nabla \varphi_s$ (S12)

φ_l = phil (electrolyte potential), φ_s = phis (electrode potential)

Where σ is the conductivity (S m^{-1}), Q is the charge source (A m^{-3}), φ is the potential (V), I is the current density. These two equations (Eqs. S9 and 10) can be modeled as secondary current distribution functions in COMSOL. The kinetic reaction to follow the anodic at WE (Eq. S11) or cathodic at CE (Eq. S12) Tafel equations in COMSOL[5].

$$i = i_0 10^{\eta/A_a} \quad \text{S13}$$

$$i = i_0 10^{-\eta/A_c} \quad \text{S14}$$

The neutral mass transfer governing equation:

$$N_i = -D_i \nabla c_i - z_i u_{m,i} F c_i \nabla \phi_l + c_i u \quad \text{S15}$$

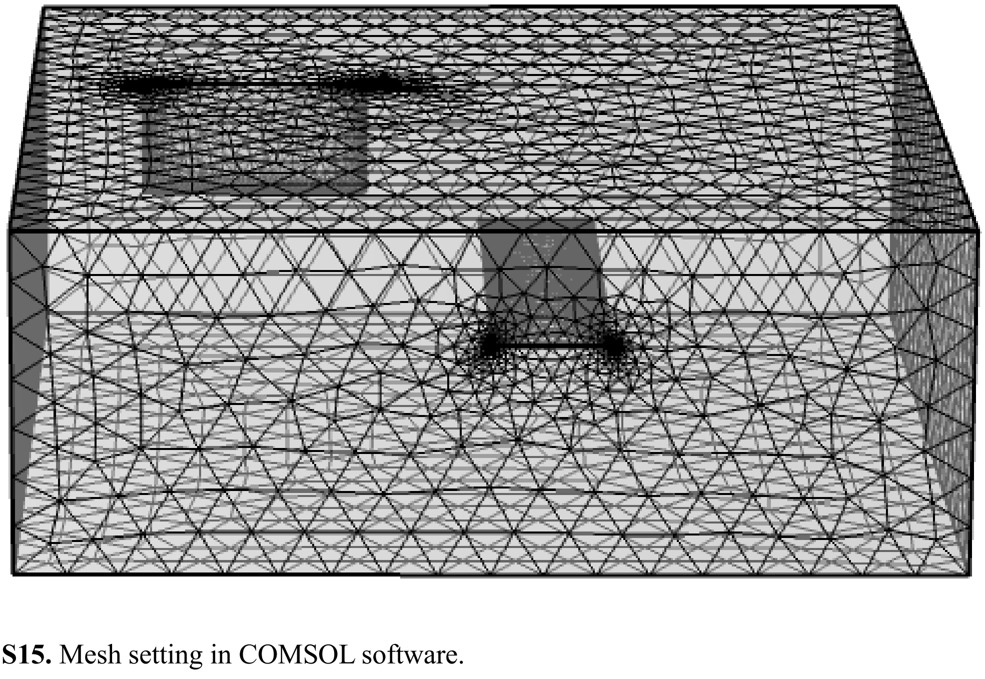

Figure S15. Mesh setting in COMSOL software.

Reference

[1] S. Li, Y. Gao, N. Li, L. Ge, X. Bu, P. Feng, *Energy & Environmental Science* **2021**, *14*, 1897-1927.

[2] Q. Deng, J. Zhao, T. Wu, G. Chen, H. A. Hansen, T. Vegge, *Journal of Catalysis* **2019**, *370*, 378-384.

[3] B. Mukherjee, *ChemistrySelect* **2021**, *6*, 609-616.

[4] E. J. Dickinson, H. Ekström, E. Fontes, *Electrochemistry communications* **2014**, *40*, 71-74.

[5] M. Kaya, N. Demir, *Fuel Cells* **2017**, *17*, 37-47.

Author Contributions

X.C., C.M. and H.P. conceived the study. X.C. and Y.H.Q. performed the experiments. X.C. and C.M. built the microscope and analyzed the data. B.X.S., W.X., S.Y.Z., L.N.Z., C.H.Y, and Q.X. advised the manuscript. X.C. and C.M. wrote the paper.
